# Scalable Ir-Doped NiFe_2_O_4_/TiO_2_ Heterojunction Anode for Decentralized Saline Wastewater Treatment and H_2_ Production

**DOI:** 10.1007/s40820-024-01542-x

**Published:** 2024-10-28

**Authors:** Sukhwa Hong, Jiseon Kim, Jaebeom Park, Sunmi Im, Michael R. Hoffmann, Kangwoo Cho

**Affiliations:** 1 Division of Environmental Science and Engineering, Pohang University of Science and Technology (POSTECH), Pohang, 790−784 Korea; 2 Linde Laboratory, California Institute of Technology, Pasadena, CA 91125 USA; 3 Institute for Convergence Research and Education in Advanced Technology (I-CREATE), Yonsei University International Campus, Incheon, 21983 Republic of Korea

**Keywords:** Wastewater electrolysis cell, Ir-doped NiFe_2_O_4_, Reactive chlorine species, Decentralized H_2_ production, On-site wastewater treatment

## Abstract

Wastewater electrolysis cells (WECs) for decentralized wastewater treatment/reuse coupled with H_2_ production can reduce the carbon footprint associated with transportation of water, waste, and energy carrier. This study reports Ir-doped NiFe_2_O_4_ (NFI, ~ 5 at% Ir) spinel layer with TiO_2_ overlayer (NFI/TiO_2_), as a scalable heterojunction anode for direct electrolysis of wastewater with circumneutral pH in a single-compartment cell. In dilute (0.1 M) NaCl solutions, the NFI/TiO_2_ marks superior activity and selectivity for chlorine evolution reaction, outperforming the benchmark IrO_2_. Robust operation in near-neutral pH was confirmed. Electroanalyses including *operando* X-ray absorption spectroscopy unveiled crucial roles of TiO_2_ which serves both as the primary site for Cl^−^ chemisorption and a protective layer for NFI as an ohmic contact. Galvanostatic electrolysis of NH_4_^+^-laden synthetic wastewater demonstrated that NFI/TiO_2_ not only achieves quasi-stoichiometric NH_4_^+^-to-N_2_ conversion, but also enhances H_2_ generation efficiency with minimal competing reactions such as reduction of dissolved oxygen and reactive chlorine. The scaled-up WEC with NFI/TiO_2_ was demonstrated for electrolysis of toilet wastewater.

**Supplementary Information:**

The online version contains supplementary material available at 10.1007/s40820-024-01542-x.

## Highlights

- Ir-doped NiFe_2_O_4_ (NFI) spinel with TiO_2_ heterojunction overlayer brought about outstanding chlorine evolution reaction in circumneutral pH.
- Electroanalyses including operando X-ray absorption spectroscopy uncovered the active role of TiO_2_ for Cl^−^ chemisorption.
- NFI/TiO_2_ anode boosted both NH_4_^+^-to-N_2_ conversion and H_2_ generation in wastewater, and the practical applicability was confirmed with scaled-up anodes and real wastewater.

**Supplementary Information:**

The online version contains supplementary material available at 10.1007/s40820-024-01542-x.

## Graphical abstract

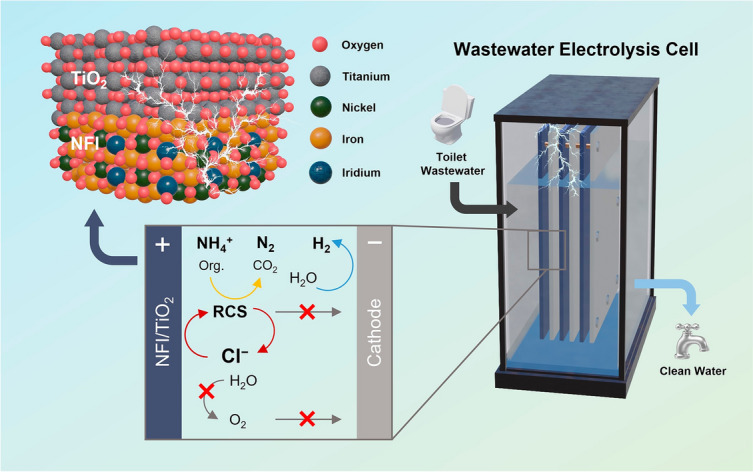

## Introduction

The current societal pursuit toward carbon neutrality would necessitate self-contained systems, ultimately to be independent on the existing water and energy grid. For example, on-site wastewater treatment and reuse are beneficial for a sustainable water cycle in adaptation to the climate change [1]. In addition, a reduction in water and waste transportation would decrease the carbon footprint [2] for sanitation and hygiene to meet the Sustainability Development Goals established by the United Nations. To this end, electrochemical oxidation processes (EOPs) have emerged as a promising way of decentralized treatment of toilet wastewater and effluent reuse [3]. While achieving adequate effluent level set by the International Organization for Standardization (*e.g*., ISO 30500 [4]), the EOPs could be advantageous with respect to ease of automation and connection with renewable energy sources (*e.g*., using photovoltaic panels) [5]. A long-term operation of a combined anaerobic digester and EOP has been demonstrated for a self-contained public toilet with a nonpotable water reuse (flushing) [6].

The chlorine evolution reaction (ClER) on electrocatalysts oxidizes chloride ion to reactive chlorine species (RCS), the core mediator to degrade aqueous organic pollutants and ammonium (NH_4_^+^) [7–9]. In particular, the efficient deammonification by the electrolytic RCS has been a subject of significant attention [10–12], which has been rarely achieved by conventional septic systems or other non-sewered sanitation systems based on biological (de)nitrification, stripping, ion exchange, and wet chemical treatments [10, 13]. Almost stoichiometric conversion of NH_4_^+^ to N_2_ by the *in situ* generated RCS without a generation of N-containing greenhouse gases (*e.g*., N_2_O, NH_3_) should be environmentally sustainable, while alleviating concerns related to NH_4_^+^ such as eutrophication and odors.

On the other hand, a distributed electrolysis of nontraditional water sources including wastewater (effluent) can be involved within the H_2_ economy [14]. A local production of deionized water by reverse osmosis is known to contribute marginally to the overall H_2_ production cost by electrolysis. However, it can compete with drinking water production in the areas with surplus renewable energy (*e.g*., desert). In this regard, contributions from Hoffmann and coworkers [15] advocate wastewater electrolysis cells (WECs) for localized conversion of renewable energy into H_2_, reducing the costs and CO_2_ emission for (waste) water treatment and transportation. A usage of separator (*e.g*., proton exchange membrane) in a direct wastewater electrolysis could bring about proliferating ohmic losses and contamination of the separator in wastewater matrix. In the single-compartment WEC, therefore, oxygen reduction reaction competes with the hydrogen evolution reaction (HER), substantially decreasing the current and energy efficiency [15]. Relatively low-grade H_2_ (< 60%) in mixture with N_2_ (from deammonification) and CO_2_ (from mineralization) can be utilized by combustion, in a decent analogy with the existing chloralkali processes that generates H_2_ as a byproduct. This approach might be more available and appropriate practice.

Nonetheless, the bottlenecks of WEC include requirements of precious element-based electrocatalysts and unsatisfactory selectivity of ClER. The current anode materials in EOPs exclusively rely on dimensional stable anode (DSA; IrTaO_x_ and RuTiO_x_) [16, 17] and boron-doped diamond (BDD) [18], unaffordable for a decentralized system. In spite of the recent developments on electrocatalysts based on earth-abundant elements (*e.g*., Ni, Fe, Co, Cu, Zn, Mo among others) [19–23], their instability in near-neutral pH required an alkalified wastewater, while inferior ClER selectivity with dominant oxygen evolution reaction (OER) ruled out a concurrent pollutants degradation during the electrolysis [24]. To this end, evidences have been presented that TiO_2_ outer layers in heterojunction with conductive Ir-based DSA could enhance both the ClER selectivity and durability [7–9], although the underlying mechanism remains ambiguous. In addition, we recently reported NiFe_2_O_4_ (NF) electrocatalysts with a tiny amount (5 mol%) of Ir doping (NFI) could enable extraordinary OER activity and stability [25]. A scaling relation between OER and ClER on (mixed) metal oxide electrocatalysts motivated us to further deploy the NFI for ClER in circumneutral pH in combination with the TiO_2_ heterojunction layer.

Within the aforementioned context, this study reports that NFI/TiO_2_ heterojunction anode (prepared by a straightforward solution casting) allows ClER activity superior to the benchmark IrO_2_ and almost absolute ClER selectivity in 0.1 M NaCl solutions. Electrolysis of NH_4_^+^-laden synthetic wastewater demonstrated that the admirable ClER metrics simultaneously enhanced the kinetics of pollutants degradation and H_2_ generation. Electroanalyses coupled with *operando* X-ray absorption spectroscopy revealed active ClER primarily on TiO_2_, while the underlying NFI served as an ohmic contact. The practical applicability was validated by a scaled-up WEC with toilet wastewater.

## Experimental Section

### Preparation of NFI/TiO_2_ Anode

**Fig. 1:**
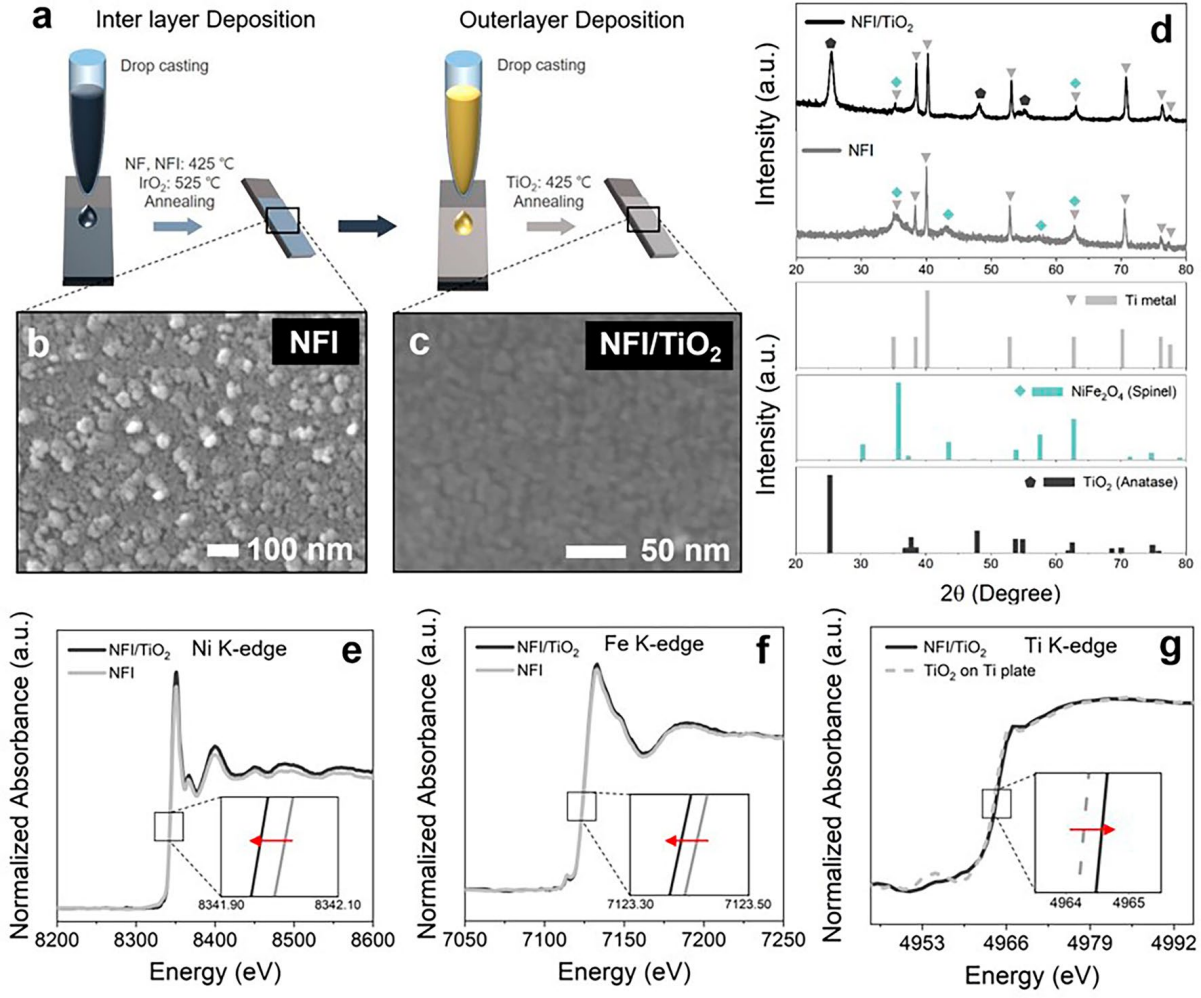
Preparation and characterization of NFI/TiO_2_ anode. **a** Schematic illustration of the synthesis procedure. **b-c** Horizontal SEM images of NFI and NFI/TiO_2_. **d** XRD profiles of NFI and NFI/TiO_2_ with references. **e–g** *Ex situ* XANES for Ni K-edge, Fe K-edge, and Ti K-edge of NFI/TiO_2_ in comparison with NFI or Ti/TiO_2_

Ti foils (Alfa Aesar, 3 × 1 cm^2^, 0.25 mm thick, 99.5% purity) underwent pretreatments to remove impurities, including SiC sandblasting, degreasing by ultrasonication in a mixed solvent (with equal volumes of ethanol, acetone, and deionized (DI) water (18.2 MΩ, Millipore)) for 0.5 h, and immersion in 10 wt% boiling oxalic acid for 0.5 h. The precursors for mixed Ni–Fe oxides were prepared using nitrate salts (Ni(NO_3_)_2_·6H_2_O and Fe(NO_3_)_3_·9H_2_O, both from Alfa Aesar in 99% purity) dissolved in DI water with 0.1 M urea, in variable molar ratios of Ni to Fe ([total metal] = 250 mM). In particular, the precursor with Ni-to-Fe ratio of 1:2 was used for NF. For IrO_2_ preparation, 250 mM H_2_IrCl_6_ was dissolved in a mixed solution with equi-volumes of ethanol, isopropanol, and 0.3 M HCl. A calculated amount of the Ir-precursor was added to the NF precursor ([Ir] = 12 mM) for the NFI. Ti-glycolate precursor for TiO_2_ layer was prepared by a peroxo-method [7, 26]. In short, 0.25 M Ti(C_4_H_9_O)_4_ was dissolved in 0.4 M glycolic acid solution by addition of concentrated H_2_O_2_, and the final pH was adjusted to be circumneutral by addition of concentrated NH_4_OH. All anodes interrogated in this study were fabricated by drop-casting (1 μL cm^−2^), drying for 15 min (80 °C), and annealing for 15 min (425 °C for NF, NFI, and TiO_2_; 525 °C for IrO_2_). This sequence was repeated up to total 6 coats which underwent final annealing for 1.5 h (Fig. 1a). A commercial BDD electrode as a control was provided by Wesco Electrode.

### Anode Characterization

The surface morphology was observed by high-resolution field emission scanning electron microscope (FE-SEM, JSM 7800F PRIME). The elemental compositions were estimated by energy-dispersive X-ray spectrometer (EDS, LN2 Free SDD type) with FE-SEM, X-ray fluorescence (ED-XRF, SII Nano technology Inc., SEA1200VX), and glow discharge spectrometry (GDS, LECO GDS850A with Radio Frequency Lamp). The crystalline structure was analyzed by X-ray diffraction (XRD, Phillips X’Pert Panalytical diffractometer) at 30 mA, 40 kV, and monochromated Cu Kα1 radiation. Raman spectra were collected by Alpha 300R (WITec) with a × 50 objective and wavelength of 488 nm using an Ar^+^ excitation source. The composition and oxidation states on surface (up to ~ 10 nm) were investigated by K-ALPHA X-ray photoelectron spectroscopy (XPS, Thermo Fisher Scientific, UK) using a monochromated Al Kα (12 kV, 72 W, 1486.6 eV, 400 μm spot size). The bulk electronic structure was interrogated by X-ray absorption spectroscopy at the 10C beamline in Pohang Accelerate Laboratory (PAL), to give X-ray absorption near-edge structure (XANES) spectra. *operando* XANES analysis proceeded with the working electrode attached to the cell window by Kapton tape, under open circuit voltage (OCV), pre-ClER, and ClER condition at a minute interval.

### Electroanalysis

A single-compartment cell (working volume: 35 mL) was used with three-electrode configuration including an anode under investigation (effective geometric area: 2 × 1 cm^2^), a Pt coil cathode (BASi), and a reference electrode. Ag/AgCl (BASi) and Hg/HgO (BASi) reference electrodes were used for electrolyte with neutral and alkaline pH, respectively. The spacing between the working and counter electrode was maintained at 0.5 cm. The measured potentials were converted to RHE scale by *E*_RHE_ = *E*_Ag/AgCl_ + 0.197 + 0.059 × pH = E_Hg/HgO_ + 0.140 + 0.059 × pH. The working electrode potential (*E*_we_) was compensated with ohmic (*iR*) drop, based on a current interruption (CI) method at 85% level. The electrochemical activity and stability were evaluated based on cyclic voltammetry (CV), linear sweep voltammetry (LSV), and chronopotentiometry using a potentiostat (VSP, BioLogic). Double-layer capacitance (C_DL_), which represents the electrochemically active surface area (ECSA), was measured by CV in a non-Faradaic potential window at variable scan rates (1 to 100 mV s^−1^) in 0.1 M NaCl (*j*_a_–*j*_c_ at 0.861 V_RHE_) and 1 M KOH (*j*_a_–*j*_c_ at 1.17 V_RHE_). The electrochemical impedance spectroscopy (EIS) estimated solution resistance (*R*_s_), films resistance (*R*_f_), and charge transfer resistance (*R*_ct_), with fitting by EC − Lab software (VSP, BioLogic). The baseline potential for EIS was 1.3 V Ag/AgCl in 0.1 M NaCl, while the sinus amplitude of 10 mV and frequency scan range of 100 kHz to 100 mHz were used. The potential of zero charge (PZC) was determined based on the potential where the capacitance was minimized [27]. The changes in capacitance at different potentials were tracked using EIS operated through a potentiostat (VSP, BioLogic). These impedance measurements, which varied with the applied potential, were taken in 0.05 M NaCl solutions, using a frequency of 150 mHz and a sinus amplitude of 5 mV. By applying 6th-order polynomial fitting to the spectra, the point where F was at its minimum was established as the PZC [27]. The Mott–Schottky (M-S) plots were obtained by EIS (sinus amplitude: 10 mV, frequency range: 10 kHz to 10 Hz, and potential range: 0 to 1 V versus reference electrode). The M-S slope from the following equation was used to comparatively evaluate the electrical conductivity [28, 29]:

1 $$\frac{1}{{C_{sc}^{2} }} = \left( {\frac{2}{{\varepsilon \varepsilon_{0} eA^{2} N_{d} }}} \right)\left[ {\left( {E_{we} - E_{FB} } \right) - \frac{kT}{e}} \right]$$

where *C*_*SC*_ is the space charge capacitance (F), *ε* is the dielectric constant, *ε*_*0*_ is the permittivity of the vacuum (8.854 × 10^−12^ F m^−1^), *e* is the elementary charge (1.602 × 10^−19^ C), *N*_*d*_ is donor density (m^−3^), *A* is active surface area (m^2^), *E*_we_ is the applied potential to the working electrode (V), *E*_*FB*_ is the flat band potential (V), *k* is Boltzmann’s constant (8.62 × 10^−5^ eV K^−1^), and *T* is the absolute temperature (K). Prior to all electroanalyses, the cell was rested in open circuit for 15 min.

### RCS Generation by Galvanostatic Bulk Electrolysis

The ClER in aqueous electrolyte generates free chlorine species including HOCl and OCl^−^ by pH-dependent hydrolysis of Cl_2_ [10]. The performance of ClER (RCS generation) was evaluated by galvanostatic electrolysis of 0.1 M NaCl solutions at variable current density (*j*, 10 to 50 mA cm^−2^). The evolution of [RCS] was periodically quantified with DPD reagents for initial 7 min, where ClO_3_^−^ or ClO_4_^−^ generations were negligible. This study used the following metrics for fair comparison of anodes [7, 8]. The CE_ClER_, EE_ClER_, and SR_ClER_ of ClER were estimated by the equations below [7, 8]:

2 $$\text{CEClER }(\%) = \frac{2VFd[RCS]}{ j A dt}$$

3 $${{EE_{ClER}}} \left( {{\text{mmol Wh}}^{-1}} \right) = \frac{V d[RCS]}{{E_{c} j A dt}} \times 3.6 \times 10^{6}$$

4 $${SR}_{ClER }(\text{mmol cm}^{-2}\text{h}^{-1}) = \frac{{\text{V}} \, {\text{d}}\text{[RCS]}}{\text{ A} \, {\text{dt}}} \times 360$$

where *V* represents the volume of the electrolyte (0.035 L), *F* is Faraday constant (96,485.3 C mol^−1^), d[RCS]/dt is RCS generation rate (M s^−1^), *j* is current density (A m^−2^), *t* is electrolysis time (s), and *E*_*c*_ is cell voltage (V).

### RCS-Mediated Wastewater Treatment Coupled with H_2_ Generation

Using the aforementioned cell, bulk galvanostatic (30 mA cm^−2^) electrolysis experiments for RCS-mediated conversion of NH_4_^+^ to N_2_ with simultaneous HER proceeded in synthetic wastewater samples. The [Cl^−^]_0_ was fixed at 0.1 M, while [NH_4_^+^]_0_ was varied ([NH_4_^+^]_0_:[Cl^−^]_0_ = 2:1, 1.5:1, 1:1, 1:1.5, 1:2, 1:3, and 1:4 in molar basis) by mixing NH_4_Cl, NaCl, and (NH_4_)_2_SO_4_. The CE of pollutants oxidation as well as CE and EE for HER were estimated by following equations [15]:

5 $${CE}(\text{pollutants oxidation}) = \frac{n V F dC}{ j A dt}$$

6 $${CE}_{HER}= \frac{\text{2 }{F}\,{Q}}{{j} \, {\text{A}}}$$

7 $${EE}_{HER}= \frac{{HHV} \,{{Q}}}{{{E}}_{\text{c}} \,{j} \,{A}}$$

where *n* is the number of electron transfer for oxidation of aqueous pollutants (3 for NH_4_^+^-to-N_2_ conversion), *dC/dt* is decreased pollutants concentration per unit electrolysis time of *t* (M s^−1^), *Q* is the observed H_2_ production rate (mol s^−1^), and HHV is higher heating value of H_2_ (78 Wh mol^−1^).

To demonstrate practical applicability, a pilot-scale WEC was manufactured in working volume of 10 L (with internal circulation). Scaled-up NFI/TiO_2_ anodes and commercial stainless steel 304 cathode were prepared in size of 35.8 × 26.6 cm^2^. Three anodes and cathodes were alternately sandwiched (with inter-electrodes distance of 1 cm) and connected to a power supply (ODA Tech, EX30-60) in monopolar configuration. The geometric surface area of the electrode module exposed to electrolyte was 0.191 m^2^. Toilet wastewater was mimicked by mixing livestock excretion (collected from Gyungju wastewater treatment plant, Korea), seawater (collected in Pohang, Korea), and tap water by volume ratio of 5:20:75. The composition of the toilet wastewater was summarized with 102 mgN L^−1^ of NH_4_^+^, 104 mgN L^−1^ of total nitrogen (TN), 580 mgO_2_ L^−1^ of chemical oxygen demand (COD), 120 NTU of turbidity, 121 mM of Cl^−^, 7.9 of pH, and 14.7 mS cm^−1^ of conductivity. The wastewater sample was subjected to electrolysis at constant current of 52.5 A (corresponding to 27.5 mA cm^−2^) for 3 h.

Quantification of anions (*e.g.*, Cl^−^, ClO_3_^−^, NO_2_^−^, and NO_3_^−^) was carried out by ion chromatography (IC, DX-120). The concentration of free chlorine and total chlorine were measured using DPD (N,N-diethyl-p-phenylenediamine) and DPD/KI reagents, respectively, based on absorbance at 530 nm in UV–Vis spectrometer (DR 3900, HACH). Combined chlorine (e.g., chloramines) was estimated from the difference between free chlorine and total chlorine [30]. TN was quantified using alkaline persulfate digestion [31] based on absorbance of nitrate at 420 nm. NH_3_-N was analyzed by salicylate method with commercial kits (NH_3_-N TNT kit, HACH) and absorbance at 610 nm [32]. [COD] was measured by dichromate digestion with colorimetric detection at 348 nm [33]. Gaseous H_2_, N_2_, and O_2_ in reactor headspace were streamed with carrier Ar gas to pass through a gas flow meter (Ritter MilliGascounter), and the composition was measured by gas chromatography with thermal conductivity detector (GC-TCD, 6890-N, Agilent Technologies). Turbidity of toilet wastewater was measured using turbidity colorimeter (HUMAS, TURBY 1000). The excitation-emission matrix (EEM) with fluorescence spectrometer (FluoroMax-4) was collected to qualitatively investigate the variations in dissolved organic matter (DOM).

## Results and Discussion

### Characterization of NFI/TiO_2_ Heterojunction Anode

The NFI/TiO_2_ anodes were fabricated through a straightforward drop-casting method (Fig. 1a). The horizontal images of FE-SEM discovered NFI nanoparticles sized in the range of 50–100 nm (Fig. 1b) which aggregated during the thermal treatment to bring about tableland and ridge morphology on the Ti substrate, as shown by EDS mapping (Fig. S1). The M-edge signals of Ir and K-edge signals of Ni, Fe, Ti, and O revealed even elemental distributions on the NFI aggregates. The EDS-based molar fraction of Ir was 5.27% for NFI, in decent agreement with 5.15% from ED-XRF analysis (Fig. S2). These values close to the precursor composition (5%) suggested homogeneous Ir doping on NFI. XRD patterns of NF and NFI powders (physically abraded from the Ti substrate) confirmed that the NiFe_2_O_4_ spinel crystalline lattice (JCPDS No. 10–0325, 2θ = 36°, 43°, and 63° corresponding to (311), (400), and (440)) [25] of NF was retained for NFI despite the Ir dopants (Fig. S3a). Raman spectra for NF and NFI (Fig. S3b) both exhibited congruence with the NiFe_2_O_4_ spinel structure of space group Fd-3 m, as affirmed with active bands including A_1g_ (symmetric stretch), E_g_ (symmetric bend), and T_2g_ (asymmetric stretch) for tetrahedral and octahedral sites [34]. This evidence substantiated the uniform doping of Ir into NF nanoparticles without insignificant structural perturbation and segregation into IrO_2_.

The SEM/EDS analysis on NFI/TiO_2_ noticed a stacked film of TiO_2_ nanoparticles sized by 10–20 nm (Fig. 1c), to allow more even deposition of TiO_2_ outer layer reducing surface tortuosity (Fig. S4). The nanoporous property of the TiO_2_ layer would allow diffusive penetration of reactants such as H_2_O, OH^−^, and Cl^−^ [26]. The XRD patterns of NFI and NFI/TiO_2_ electrodes (Fig. 1d) were dominated by signals from the Ti metal substrate (JCPDS No. 44–1294, 2θ = 35°, 38°, 40°, 52°, 62°, 70°, 76°, and 77°) that largely overlapped with those of spinel NiFe_2_O_4_ peaks. NFI/TiO_2_ showed additional diffraction peaks from anatase TiO_2_ (JCPDS No. 21–1272, 2θ = 25° and 48° corresponding to (101) and (200), respectively). These evidences characterized the NFI/TiO_2_ as Ir-doped NiFe_2_O_4_ in heterojunction with nanoporous TiO_2_ layer. A GDS analysis (Fig. S5) estimated the thickness of TiO_2_ and NFI to be ~ 250 nm and ~ 2.75 µm, respectively.

XPS for NF and NFI (Fig. S6) clarified partial charge transfer from Ni and Fe to the Ir dopants. The fractions of Ni^3+^ and Fe^3+^ from deconvoluted Ni 2*p*_3/2_ (854.5 eV for Ni^2+^ and 856 eV for Ni^3+^ with two satellite peaks at 861 and 865 eV) [35] and Fe 2*p*_3/2_ [36] (710 eV for Fe^2+^and 711.5 eV for Fe^3+^) photoelectron spectra were elevated in NFI, whereas the binding energy of Ir 4*f* peak was between those of Ir^4+^ (61.8 eV) and Ir^0^ (60.9 eV) [37]. The deconvolution of O 1*s* spectra noted escalated fraction of oxygen vacancy upon the Ir doping. These observations were in agreement with the prior report on NFI coated on Ni foam [25]. On the other hand, the Ti 2*p* photoelectron spectra for NF/TiO_2_ and NFI/TiO_2_ both indicated a partial oxidation of Ti (Fig. S7), in comparison with the TiO_2_ layers directly coated on the Ti substrate (Ti/TiO_2_). However, the concurrent shifts in electronic structure of the underlying layers were intangible due to the limited analytical depth of XPS. To this end, XANES unambiguously informed on the electronic interaction across the heterojunction, based on the edge position of individual metal components at the half-maximum intensity to represent the oxidation state. The Ti K-edge position of NFI/TiO_2_ was positively shifted compared to Ti/TiO_2_ (Fig. 1f), whereas both Ni and Fe K-edge region absorbance spectra for NFI/TiO_2_ suggested decreased valency compared to NFI (Fig. 1e, f). The *ex situ* XANES thus provides compelling evidence of charge transfer from the outer TiO_2_ to the underlying NFI across the interface.

### Electrocatalytic Behaviors of NFI/TiO_2_ Heterojunction Anode

Given the scaling relation between OER and ClER intermediates for (mixed) metal oxide electrocatalysts [38], screening electrocatalysts in terms of OER activity could be a precedent step to employ the OER intermediates as ClER center [26]. Due to a composition-dependent instability of Ni_x_Fe_1-x_O_y_ electrocatalysts in acidic-to-neutral pH, moreover, it was inevitable to evaluate them in alkaline electrolyte where OER would overwhelm ClER. LSV curves in 1 M KOH confirmed extraordinary OER activity of NiFe_2_O_4_ spinel oxide substantially outperforming the other Ni_x_Fe_1-x_O_y_ compositions (x = 0, 0.2, 0.5, 0.67, 0.8, and 1) identically synthesized by dip coating (Fig. S8) [26, 39]. Mixing Ir within the NiFe_2_O_4_ precursor at variable atomic ratio (0, 1%, 3%, 5%, 7%, and 10%) enhanced OER activity up to 5% Ir on the modified electrocatalysts, judging from overpotential (*η*) at 10 mA cm^−2^ (Fig. S9). Further elevation in Ir contents marginally influenced the current wave, in compatible with the previous report [25].

**Fig. 2:**
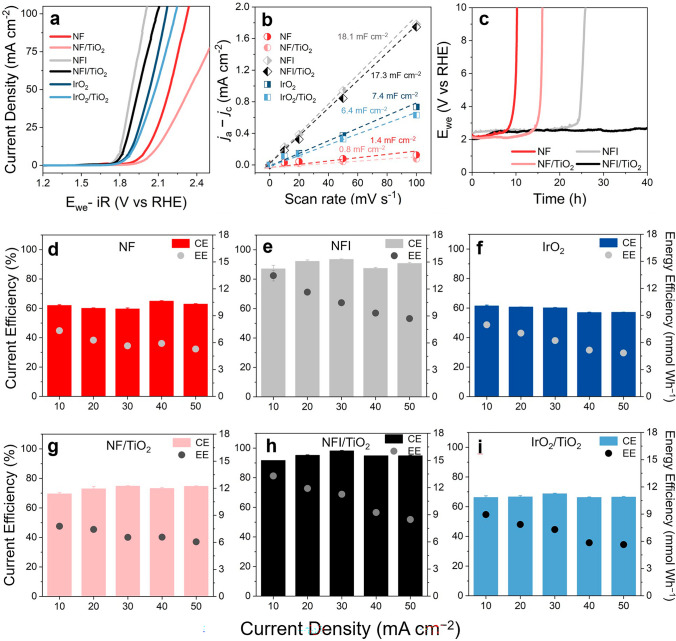
Electrochemical performances. **a** LSV curves (scan rate: 10 mV s^−1^) with 85% *iR* correction. **b** Capacitive *j*_a_–*j*_c_ versus scan rate from CV (potential range: 0–0.5 V vs. Ag/AgCl, scan rate: 10, 20, 50, and 100 mV s^−1^) for NFI, NFI/TiO_2_, NF, NF/TiO, IrO_2_, and IrO_2_/TiO_2_ electrocatalysts in 100 mM NaCl. **c** Chrono-potentiometric profile for long-term stability test of NFI, NFI/TiO_2_, NF, NF/TiO_2_ in 0.5 M NaClO_4_. **d-i** CE_ClER_ and EE_ClER_ during galvanostatic electrolysis of 0.1 M NaCl solutions for NF, NFI IrO_2_, NF/TiO_2_, NFI/TiO_2_, and IrO_2_/TiO_2_

Armed with the supreme OER activity of NFI (5% Ir-doped NiFe_2_O_4_), the electrochemical performances of NFI and NFI/TiO_2_ were assessed in 0.1 M NaCl electrolyte with circumneutral pH, and compared with NF and IrO_2_ with or without the TiO_2_ overlayer. It should be noted that ClER and OER would occur in parallel in this experimental condition. The XRD for the control group samples confirmed crystallinity of spinel NiFe_2_O_4_, rutile IrO_2_ (JCPDS No. 15–870), and anatase TiO_2_. (Fig. S10). The voltammograms (Fig. 2a) estimated required potentials at 10 mA cm^−2^ to be 2.00, 2.07, 1.81, 1.83, 1.88, and 1.90 V versus reversible hydrogen electrode (RHE) for NF, NF/TiO_2_, NFI, NFI/TiO_2_, IrO_2_, and IrO_2_/TiO_2_, respectively. The NFI exhibited the most facile charge transfer kinetics, even outperforming the benchmarked IrO_2_. Although the TiO_2_ overlayer moderately lowered the anodic wave, NFI/TiO_2_ still marked superior activity compared to the IrO_2_. The C_DL_ was measured by plotting charging current density (*j*_a_−*j*_c_) as linear functions of scan rate (Figs. 2b and S11). The *C*_DL_ value, as a surrogate of ECSA, showed analogous trend with the LSV. Nyquist plot from EIS disentangled *R*_f_ and *R*_ct_ [40], as shown in Fig. S12. The IrO_2_ exhibited singular semicircle owing to the conductor-like property, whereas the *R*_f_ was noted for NF and NFI by additional semicircles in lower frequency ranges. The *R*_ct_ based on diameter of the higher frequency semicircles agreed with the activity trends, while the TiO_2_ overlayers substantially increased the *R*_f_ for the heterojunction anodes. Therefore, the moderate current reduction by the TiO_2_ layer was ascribed to resistance to charge migration (due to an inferior electrical conductivity of TiO_2_) and/or pore diffusion through the nanoporous film that was incompletely compensated by the CI method.

The overall activity trends were maintained in voltammograms obtained in 1 M KOH (Fig. S13), because of the scaling relation between adsorption energy for intermediates of OER (*OOH) and ClER (*OCl) on metal oxide electrocatalysts [7, 8]. The OER *η* of NFI (330 mV) at 10 mA cm^−2^ was lower than NF and IrO_2_. We previously presented evidences that Ir doping on NiFe_2_O_4_ could shift the active motif from Fe–O–Fe to Ni–O–Fe to concurrently escalate ECSA and intrinsic OER activity of NF [25]. If the porous TiO_2_ layers were electrochemically inert, on the other hand, the reduction of ECSA by the TiO_2_ deposition would be independent on the electrolyte while the mass transport resistance through the TiO_2_ film could be alleviated in 1 M KOH. However, the TiO_2_ layer reduced the *C*_DL_ value (Fig. S14) of NFI more significantly in 1 M KOH compared to those in 0.1 M NaCl. It implicitly elucidated an active electrocatalytic roles of TiO_2_ for ClER. Mott–Schottky slopes both in 1 M KOH and 0.1 M NaCl (Fig. S15) further revealed substantially elevated donor density and electrical conductivity for NFI, compared to NF. The observed p-type property of NiFe_2_O_4_ [41] rationalized the electron withdrawing from the TiO_2_ (well-known n-type semiconductor) through p-n heterojunction (Fig. S7) [42, 43]. The superior conductivity of NFI should be advantageous as an ohmic contact to reduce the energy barrier for charge transfer from TiO_2_ under a forward bias on the anode.

The anodic bias coupled with the generations of RCS and protons in the anode vicinity would cause dissolutions of Ni and Fe [44, 45], which could be accelerated as bulk pH decreases. The long-term stability tests were performed in 0.5 M NaClO_4_ solution (pH ~ 7) under 30 mA cm^−2^, where the durability of NFI/TiO_2_ (in terms of potential variation over 40 h) clearly outperformed NFI, NF and NF/TiO_2_ (Fig. 2c). Repetitive CV further demonstrated the superior stability of NFI/TiO_2_ with negligible activity loss during 100 cycles, compared to NF/TiO_2_ (Fig. S16). Judging from the well-known stability of TiO_2_ in wide potential and pH windows in Pourbaix diagram [46], the varied durability depending on the underlying layer demonstrated penetration of electrolyte through pores and pinholes of the TiO_2_ layer. An adsorbate evolution mechanism prevalent on NF and NFI was reported to allow greater service life than other Ni_x_Fe_1-x_O_y_ electrocatalysts based on lattice oxygen mediated mechanism, while the Ir dopants could further increase the required energy for atomic defect formation (dissolution) [25]. In addition, the TiO_2_ overlayer could alleviate the diffusion of dissolved components from the buried NF or NFI toward the bulk phase, further reinforcing the stability [26].

The figures-of-merit for ClER were comparatively evaluated in 0.1 M NaCl solutions (Fig. 2d-i) at various current densities (10 to 50 mA cm^−2^). The galvanostatic regime has been widely deployed for industrial processes owing to more straightforward scaling-up and process control than potentiostatic one. In this condition, the specific ClER rate (SR_ClER_, Fig. S17) was merely proportional to current efficiency (CE_ClER_). The CE_ClER_, the core metric of ClER selectivity, was insignificantly influenced by the applied current densities in this experimental range. This is presumably due to a lack of diffusion limitation for Cl^−^, which would allow a flexible operation of EOPs upon a fluctuation of influent wastewater. The average of CE_ClER_ of NF, NF/TiO_2_, NFI, NFI/TiO_2_, IrO_2_, and IrO_2_/TiO_2_ was calculated to be 61.9%, 73.1%, 90.2%, 95.0%, 59.3%, and 66.9%, respectively (Fig. S18). The NFI itself marked CE_ClER_ exceeded 90% which was further increased by TiO_2_. Comparably inferior ClER selectivity of NF and IrO_2_ (~ 60% of CE_ClER_) was evidently improved by the TiO_2_ overlayers, in agreement with the previous reports [7, 8, 26]. The exhibited selectivity trend would be associated with surface properties such as O-binding strength [38]. This conjecture was interrogated using PZC, an experimental descriptor for the surface charge density [27]. Specifically, the greater PZC would correspond to the less propensity to lose electron and the lower bond strength with electrophilic *O [47]. A recent theoretical study claimed a fair correlation between the PZC and binding energy of *OH [48]. Figure S19 portrays a weak positive relation between PZC (raw data for PZC determination in Fig. S20) and CE_ClER_. The influences of specific adsorption and space charge capacitance would account for the deviations from an ideal linearity. The Ir doping on NF substantially elevated the PZC of NFI, which conformed to the escalated oxidation states of Ni and Fe in Ni–O–Fe motifs (Fig. S6). In other words, the weakened binding of *OH could facilitate reaction with Cl^−^, to rationalize the greater CE_ClER_ than NF. The TiO_2_ overlayers further increased the PZC of NF and NFI to account for the moderate enhancement in ClER efficiency. Considering the strong electronic interaction across the junction (Fig. S7), in analogy, this finding also suggested active involvement of TiO_2_ for ClER. If the buried NF or NFI served as the primary ClER sites, the charge withdrawing from the TiO_2_ overlayer could strengthen the *OH binding to bring about a reduced CE_ClER_.

The energy efficiency (EE_ClER_) in terms of molar amount of RCS per unit energy input should depend both on activity (cell voltage at the given *j*) and selectivity (CE_ClER_) [7, 26]. Unlike the CE_ClER_, EE_ClER_ value justly increased for the smaller *j* (cell voltage). The TiO_2_ overlayers gave ambivalent effects on EE_ClER_, by enhancing CE_ClER_ in trade-off by increasing the ohmic loss and cell voltage. Accordingly, beneficial improvements in EE_ClER_ by the outer TiO_2_ layer were noted for NF/TiO_2_ and IrO_2_/TiO_2_, whereas increases in EE_ClER_ for NFI/TiO_2_ were limitedly observed only at 20 and 30 mA cm^−2^ due to high ClER selectivity of NFI itself. Consequently, NFI/TiO_2_ recorded the supreme ClER performance and stability with respect to all figures-of-merit under the interrogation conditions. The aforementioned electroanalyses collectively demonstrated that a tiny amount of Ir dopants could boost the ECSA, electrical conductivity, and affinity to Cl^−^ chemisorption. They in-turn contributed to the outstanding intrinsic ClER activity of NFI, as a promising candidate to replace the precious IrO_2_ electrocatalysts. The NFI/TiO_2_ architecture further enabled more exceptional RCS generation efficacy, while the protective TiO_2_ heterojunction layer played a pivotal role to elongate the durability during ClER in near-neutral pH.

### ClER Mechanism of NFI/TiO_2_ Heterojunction Anode

**Fig. 3:**
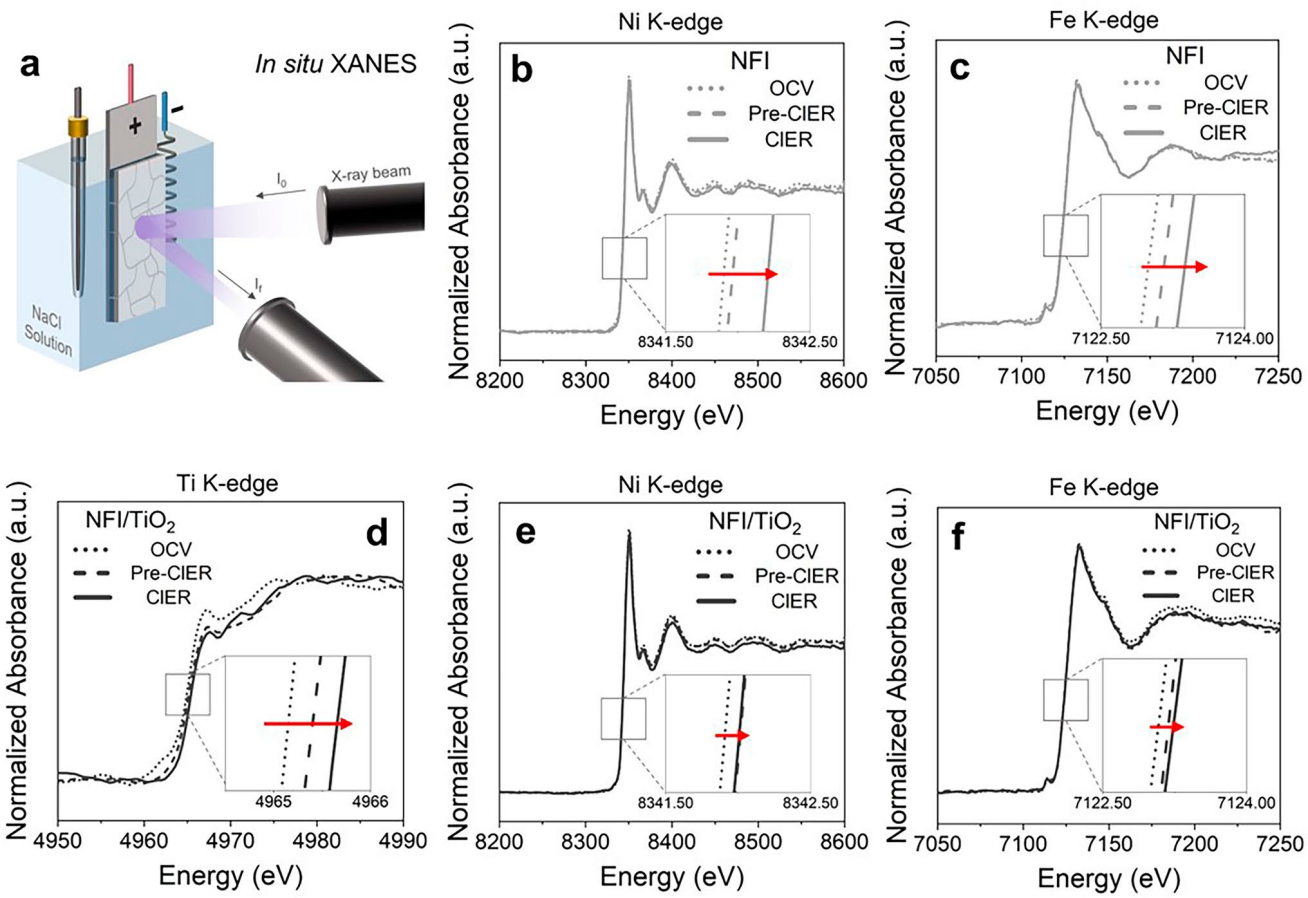
The *operando* XANES analysis. **a** The schematic illustration of the setup. **b-c** Normalized XANES spectra in Ni K-edge and Fe K-edge for NFI. **d-f** Normalized XANES spectra in Ti K-edge, Ni K-edge, and Fe K-edge for NFI/TiO_2_. The spectra were collected under OCV, pre-ClER at 1 mA cm^−2^, and ClER at 3 mA cm^−2^ in 100 mM NaCl

Our prior reports have demonstrated selective ClER in dilute (< 0.1 M) NaCl on dual-layer anodes which comprised of outer TiO_2_ layer in heterojunction with either Ir(Ta)O_x_ or NiFeO_x_ [7, 8, 26, 49]. Nevertheless, the precise active site for ClER remained equivocal for these anodes. The electroanalyses in this study including the description of CE_ClER_ by PZC suggested active participation of TiO_2_ overlayers in the ClER. In order to obtain conclusive evidences regarding the Cl^−^ adsorption site for NFI/TiO_2_, this study utilized *in situ* XANES analysis that is a powerful tool to monitor changes in the valence state of active elements. The spectra were gained under OCV, pre-ClER at 1 mA cm^−2^ (capacitive current region), and ClER at 3 mA cm^−2^. The relatively low *j* could avoid the noise by Cl_2_ gas on the anode surface [25, 50]. Figure 3 represents normalized absorbance signals in Ni, Fe, and Ti K-edge collected from NFI and NFI/TiO_2_. The Ni and Fe K-edge position for NFI markedly showed blue shifts (Fig. 3b, c) monotonically along with the elevated bias from OCV to ClER regime, nominating the Ni–O–Fe motif to be the active site [26]. On the contrary, the valency changes for both Ni and Fe on NFI/TiO_2_ were relatively insignificant upon the transition from pre-ClER to ClER regime (Fig. 3e, f), whereas a prominent shift of Ti K-edge was clearly observed (Fig. 3d). It clearly unraveled that the upper TiO_2_ would serve as the active ClER sites, while the underlying NFI transformed upon the bias (*e.g*., Ni–Fe oxyhydroxide with elevated electrical conductivity) would function as the ohmic contact for charge migration. The biased hydrated TiO_2_ could form surface *OH as the predominant intermediate conducive to the chemisorption of Cl^–^. The effective electron withdrawing by NFI across the p–n junction (Fig. S7) would further facilitate the initial discharge of Cl^–^ (the presumed rate determining step of ClER) on the charge-deficient TiO_2_. In addition, the alleviated oxidation of Ni and Fe could rationalize the elongated service life for NFI/TiO_2_ (Fig. 2c).

The roles of NFI and TiO_2_ in parallel ClER and OER were further elucidated by LSVs performed either in 0.5 M Na_2_SO_4_ (for exclusive OER) or 0.5 M NaCl (for predominant ClER as indicated by Fig. 3) electrolyte in pH ~ 7. As shown in Fig. S21, the anodic waves for OER (in Na_2_SO_4_) and ClER (in NaCl) on NFI were comparable up to 100 mA cm^−2^. The TiO_2_ overcoats diminished the activities in both electrolytes, but the *j* values on NFI/TiO_2_ in 0.5 M NaCl exceeded those in Na_2_SO_4_ electrolyte from the onset to 2.1 V RHE. This noteworthy observation also supported alteration of ClER site from NFI to TiO_2_ for the heterojunction anode. The superior ClER in relatively low potentials (< 2.1 V RHE) could attribute to favorable binding of Cl^−^ to TiO_2_. Above this potential, however, the ClER on NFI/TiO_2_ was more sluggish than the OER due to pronounced diffusion limitation for Cl^−^ through the TiO_2_ film at the elevated *j*. Figure S22 illustrates the CVs in 1 M KOH + 0.1 M K_3_Fe(CN)_6_ solution. The clearly defined reversible redox peaks of Fe(CN)_6_^3+/2+^ on NF and NFI were dramatically attenuated with the presence of TiO_2_ overlayer, because of a rejected diffusion of the molecular anion through the pores. Therefore, the active TiO_2_ for ClER was speculated to be located in the vicinity of the interface with NFI, presumably in association with the strong interaction with NFI presumably by thermal interdiffusion of metallic components across the junction [7].

### Electrochemical Deammonification Coupled with Molecular H_2_ Production

**Fig. 4:**
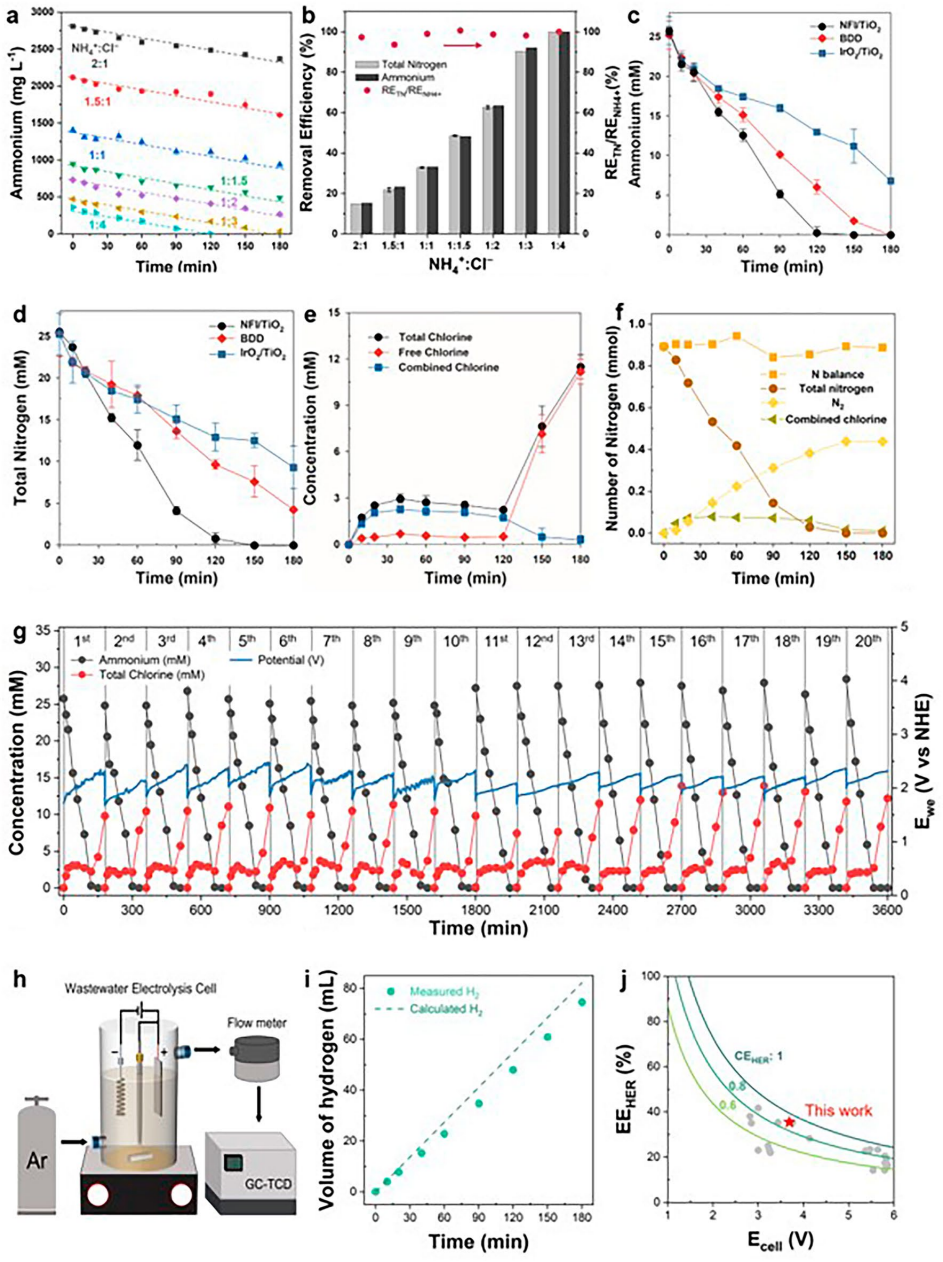
RCS-mediated electrochemical deammonification coupled with H_2_ production. **a-b** Concentration profiles of NH_4_^+^ and removal efficiency of TN and NH_4_^+^ for NFI/TiO_2_ with variable [NH_4_^+^]_0_ (25–200 mM). **c-d** Concentration profiles of NH_4_^+^ and TN for NFI/TiO_2_ in comparison with BDD and IrO_2_/TiO_2_. **e–f** Concentration profiles of free, combined, and total chlorine with N balance calculated by the sum of TN, N_2_ gas, and combined chlorine for NFI/TiO_2_. **g** Repeated batch degradations of NH_4_^+^ with NFI/TiO_2_. **h** Schematic illustration for H_2_ quantification. **i-j** Metrics in terms of CE_HER_ and EE_HER_ in comparison with theoretical values (solid lines for CE_HER_ of 1, 0.8, and 0.6) and reported values in literature (gray circle) [15, 51–53]. Galvanostatic (30 mA cm^−2^) electrolysis was performed with NH_4_^+^-laden synthetic wastewater ([NH_4_^+^]_0_ = 25 mM for **c**-**j**, [Cl^−^]_0_ = 100 mM)

The RCS-mediated NH_4_^+^ degradation experiments by NFI/TiO_2_ anode were conducted with varying [NH_4_^+^]_0_ (molar ratio of NH_4_^+^ to Cl^−^ from 0.25 to 2), at fixed [Cl^−^]_0_ of 0.1 M and *j* of 30 mA cm^−2^. As depicted in Fig. 4a, the NH_4_^+^-N conversion followed apparent zero-order kinetics with superimposable rate constants irrespective of [NH_4_^+^]_0_ (~ 10 mM h^−1^ on average), in consistent with previous reports [54]. Regardless of the initial ratios, the ammonium removal efficiency (RE) of NH_4_^+^ was nearly identical to those for TN, indicating that NH_4_^+^ was predominantly converted to gaseous N_2_ (Fig. 4b). Based on the consistent degradation kinetics, the ratio of [NH_4_^+^]_0_ to [Cl^−^]_0_ was fixed at 0.24 for further experiments to monitor the dynamic evolutions of reaction intermediate species during the galvanostatic (30 mA cm^−2^) deammonification for 3 h. The performance of NFI/TiO_2_ was compared with IrO_2_/TiO_2_ and BDD, as benchmark anodes for water treatment. Electrophilic attacks of RCS to ammonia produce chloramines that are eventually transformed to N_2_. The mechanism simplified by the following equations is widely known as breakpoint chlorination in water treatment [30].

8 $${\text{NH}}_{{3}} + {\text{ HOCl }} \to {\text{ NH}}_{{2}} {\text{Cl }} + {\text{ H}}_{{2}} {\text{O}}$$

9 $${\text{NH}}_{{2}} {\text{Cl }} + {\text{ HOCl }} \to {\text{ NHCl}}_{{2}} + {\text{ H}}_{{2}} {\text{O}}$$

10 $${\text{NH}}_{{2}} {\text{Cl }} + {\text{ NHCl}}_{{2}} \to {\text{ N}}_{{2}} + {\text{ 3H}}^{ + } + {\text{ Cl}}^{ - }$$

As shown in Fig. 4c, the NH_4_^+^ degradation rates were in the order of NFI/TiO_2_ > BDD > IrO_2_/TiO_2_, in general agreement with the ClER activity. The metrics for BDD in 100 mM NaCl was quantified with CE_ClER_ of 61.5% and EE_ClER_ of 3.94 mmol Wh^−1^. Furthermore, NFI/TiO_2_ brought about comparable profiles for [TN] and [NH_4_^+^], whereas the TN decay rates were retarded for the others, more markedly for BDD (Fig. 4d) due to formation of nitrite ions (NO_2_^−^) measured up to 5 mM (Fig. S23). Formation of nitrate ions (NO_3_^−^) was always negligible in this experimental condition for all anodes. Albeit the BDD has been extensively deployed for water treatment owing to effective utilization of bound hydroxyl radical [55, 56], the mediated TN removal was incomplete due to the hydroxyl radical mediated oxidation to NO_2_^−^ as a byproduct. The profiles of free/combined chlorine concentrations with NFI/TiO_2_ (Fig. 4e) confirmed the breakpoint chlorination mechanism; a buildup of free chlorine initiated at ~ 2 h of electrolysis which extinguished NH_4_^+^. The stoichiometric NH_4_^+^-to-N_2_ conversion by NFI/TiO_2_ was additionally supported by almost consistent N balance by the sum of measured gaseous N_2_, aqueous TN, and combined chlorine (Fig. 4f). In comparison, the [free chlorine] was in quasi-steady states for IrO_2_/TiO_2_ and BDD up to 3 h (Fig. S24), in compatible with the incomplete conversion of NH_4_^+^. The generation of oxynitrogen anions during the breakpoint chlorination could be invigorated by an elevation in relative RCS dosage [57]. Thus, intrinsically continuous and distributed feed of RCS in electrochemical chlorination would be beneficial for an ideal NH_4_^+^-to-N_2_ conversion with minimal byproducts generation. As a confirmation, a batch injection of 100 mM of NaOCl (comparable with the total RCS generated by NFI/TiO_2_ for 3 h) to the synthetic wastewater brought about rapid exhaustion of NH_4_^+^ within 15 min, but the TN decay was markedly retarded to be incomplete after 3 h. (Fig. S25). Consequently, the selective ClER on NFI/TiO_2_ effectively led to more facile achievement of breakpoint than the benchmark anodes, while far lower steady-state [RCS] compared to a chemical chlorination prevented oxyanion byproducts (NO_2_^−^ and NO_3_^−^) formation.

For all [NH_4_^+^]_0_ conditions, the ultimate concentrations of [NO_2_^−^] and [NO_3_^−^] in electrolyte were negligible (Fig. S26) to maintain RE for [TN]/[NH_4_^+^] near unity for NFI/TiO_2_ (Fig. 4b). These observations suggested that the heterogeneous charge transfer (ClER) would be rate-limiting under our galvanostatic condition [16, 58], i.e., nucleophilic attack of RCS to NH_3_ (Eq. 8) and chemical reactions among chloramines (Eqs. 9 and 10) were more facile than the ClER [16]. Regarding the mass balance of Cl, on the other hand, monotonic declines of [Cl^−^] was noted with an increasing rate as [NH_4_^+^]_0_ decreased (Fig. S26). Since oxychlorine anions (e.g., ClO_3_^−^ and ClO_4_^−^) were always negligible, likely owing to the low [RCS] before the breakpoint, the reduced [Cl^−^] would be ascribed to a volatilization of Cl_2_ gas. The general CE of NH_4_^+^-to-N_2_ conversion (3e^−^ transfer) at [NH_4_^+^]/[Cl^−^] of 0.25 was 58.6% after 2 h of electrolysis (breakpoint as indicated by Fig. S27), being far lower than the CE_ClER_ measured in 0.1 M NaCl. However, the reduced [Cl^−^] accounted for CE of 43.8% (assuming 2e^−^ transfer to RCS) to roughly close the charge balance.

In order to confirm the stability of NFI/TiO_2_ anode during the water treatment, the batch NH_4_^+^ degradation experiment was repeated up to 20 cycles (Fig. 4g). The efficacy of the RCS-mediated deammonification was maintained without a significant variation based on the pseudo zero-order rate constants of NH_4_^+^ abatement ranging from 11.1 to 12.8 mM h^−1^.The typical profiles of total/free chlorine in breakpoint chlorination were consistently reproduced as well (Fig. S28). The anodic potential moderately increased within a cycle, which was ascribed to the reduced electrical conductivity by deammonification since it was recovered at the beginning of the subsequent cycle. After the sequencing batch cycles, the used NFI/TiO_2_ retained crystalline integrity of spinel NiFe_2_O_4_ and anatase TiO_2_ structure, as revealed by XRD (Fig. S29). We additionally performed XPS and Raman spectroscopy analyses on the used NFI/TiO_2_ samples to evaluate the stability of the heterojunction anode. The analytical depth of XPS is typically 5–10 nm to target the surface [59]. As expected, the Ni and Fe 2*p* peaks in NFI/TiO_2_ displayed significantly reduced intensities compared to those in NFI, which can be attributed to the limited amount of Ni and Fe that migrated to the surface owing to a thermal diffusion during the annealing (Fig. S30). Nevertheless, these peaks were well-preserved after the extended electrolysis (Fig. S31a, b). Furthermore, both photoelectron spectra and Raman spectra of NFI/TiO_2_ before and after electrolysis reveals that the signals related to the TiO_2_ outer layer were consistent after the electrolysis. (Figs. S31c and S32). Consequently, NFI/TiO_2_ anode proved a long-term stability during electrolysis of wastewater with near-neutral pH.

Figure 4h illustrates the quantification method of H_2_ generation from the undivided WEC with NFI/TiO_2_ anode. Headspace of the gas-sealed reactor was connected to a flow meter, while the composition was measured by GC-TCD. Figure 4i presents the observed rate of H_2_ production during the galvanostatic electrolysis of the synthetic wastewater. The current efficiency for HER is an important figure-of-merit, together with energy efficiency, in the wastewater electrolysis to compare the energy conversion reaction selectivity [12, 15]. The CE_HER_ ranged 85%–90% during the course of electrolysis, averaged to 85.8% (Fig. S33). A portion of passed charge unused for the HER could be dissipated by undesired reactions, such as reduction of combined chlorine, and oxyanions (*e.g*., NO_x_^−^, ClO_x_^−^) on the Pt cathode, albeit quantification of individual side reaction was infeasible in the undivided cell. Concurrently, the EE_HER_ (representing the conversion efficiency of electric energy to H_2_) was averaged to 35.4% during the operation at 30 mA cm^−2^. The observed metrics for CE_HER_ and EE_HER_ outweighed prior reports (CE_HER_ < 80% and EE_HER_ < 23% at *j* > 20 mA cm^−2^) regarding the wastewater electrolysis with IrO_2_ based anodes and variable compositions of wastewater (Fig. 4j) [15, 51–53]. The produced gas mixture during the initial 2 h of electrolysis primarily consisted of 2.0 mmol H_2_ (83%) and 0.44 mmol N_2_ (17%).

The attenuated OER on the NFI/TiO_2_ anode could lead to negligible oxygen reduction reaction, in-turn elevating the CE_HER_. Gaseous [O_2_] in the reactor headspace was indeed below the detection limit. In addition, the facile quenching of RCS by NH_4_^+^ would minimize the chlorine reduction reaction; *i.e*., RCS-mediated oxidation of electron donating pollutants allowed selective HER even in membrane-less configuration. Therefore, the quasi-absolute selectivity for ClER on NFI/TiO_2_ could intensify the synergism in bifunctional WEC for water treatment coupled with H_2_ generation. On the other hand, the CE_HER_ and *E*_cell_ exclusively determine the EE_HER_ (Fig. 4j) [15], while the voltage loss would be governed by the anodic *η* and ohmic resistance. Consequently, the admirable electrocatalytic ClER activity of NFI/TiO_2_ also contributed to the enhanced EE_HER_. It is worth mentioning that the marked EE_HER_ value in this study can be easily raised by lowering the *E*_*c*_ (*j*), which reduces the *iR* loss but inevitably retards the ClER and mediated pollutants removal. This apparent trade-off relations between the rate of pollutants removal and energy conversion efficiency have been noted previously [15], highlighting the importance of process engineering. In addition, the EE_HER_ can be escalated with an increasing electrical conductivity of wastewater, by mixing with seawater as an example, which deserves further research.

### Scaled-Up Application for Toilet Wastewater Treatment

**Fig. 5:**
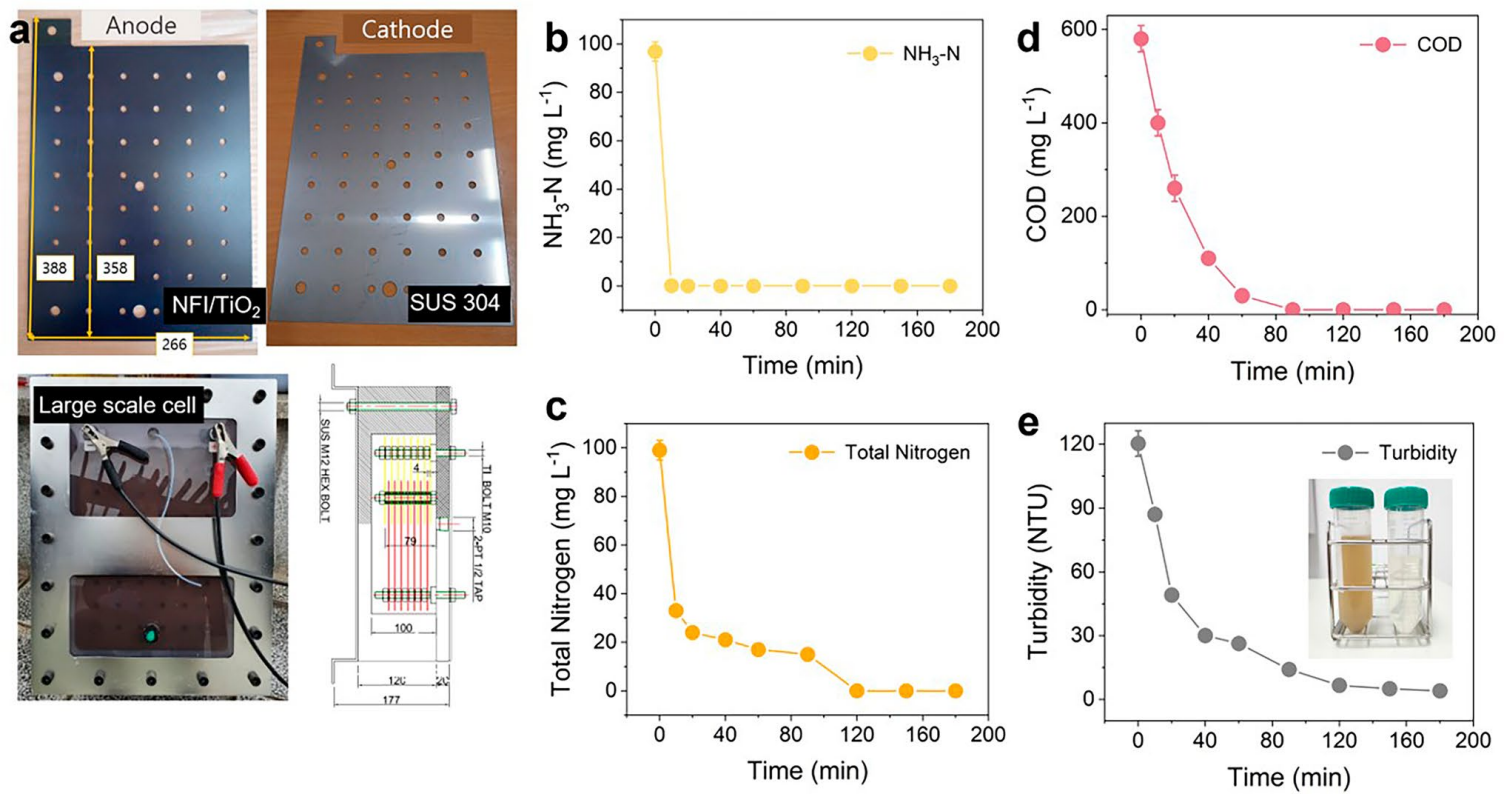
Demonstration of a scaled-up WEC with toilet wastewater. **a** Scaled-up NFI/TiO_2_ anode, stainless steel 304 cathode, and WEC (effective volume of 10 L) with a sandwich module. **b-e** Concentration profiles of NH_3_-N, TN, COD, and turbidity. The inset in **e** shows photographs of influent (left) and effluent (right)

For demonstration of practical applicability, scaled-up NFI/TiO_2_ anodes and electrolysis cell (effective volume of 10 L) were fabricated as shown in Fig. 5a. It should be mentioned that our straightforward dip coating and thermal decomposition methods for NFI/TiO_2_ were amenable for the scaling. In order to avoid uneven coating and current distribution, multiple anodes were matched with commercial AISI 304 stainless steel cathodes in a sandwich module with total surface area of 0.286 m^2^ (submerged area: 0.191 m^2^). The cost-effectiveness and moderately efficient HER property could rationalize the deployment of stainless steel [15]. Figure 5b–e depicts the profiles of principal pollutants within a cycle of sequencing batch operation at galvanostatic condition of 52.5 A (27.5 mA cm^−2^). The pilot WEC exhibited eminent removal efficiency for COD and turbidity, due to well-known reactivity of the electrolytic RCS toward organic compounds in wastewater. The EEM before and after treatment (Fig. S34) additionally showed evident reduction of humic-like substances in the DOM, as represented by the peak centered at 430/360 nm [60]. The influent NH_4_^+^ was completely eliminated within 20 min by the swift reactions with RCS, whereas the concurrent decline of [TN] became more sluggish after 20 min to demand ~ 2 h for full annihilation of TN. It was presumably because the chlorination of monochloramine to dichloramine (Eq. 9) was retarded due to competition for RCS with organic compounds and monochloramine. A formation of organic chloramines from chlorination of organic nitrogen species (e.g., protein) in wastewater could not be ruled out as well. The CE calculated for initial 20 min was 61.3% for COD oxidation and 26.9% for TN conversion, being comparable with CE_ClER_ measured in 0.1 M NaCl (86%). It corroborated that the exceptional ClER on NFI/TiO_2_ was maintained in the scaled-up process. The total CE for pollutants oxidation in real wastewater exceeded the estimates for the synthetic one with NH_4_^+^ only, because more abundant organic electron donors minimized the volatilization of unreacted Cl_2_. Additionally, our wastewater electrolysis cell with NFI/TiO_2_ anode and toilet wastewater achieved superior energy efficiency for removal of COD, TN, and NH_4_^+^-N, compared to previous electrooxidation processes using precious metal-based anodes (Table S1).

The H_2_ generation constitutes a crucial advantage of WEC for energy storage coupled with wastewater treatment, when powered by renewable energy sources. The importance of decentralized H_2_ production to reduce carbon footprint has been demonstrated recently by a life cycle analysis, primarily owing to the reduced CO_2_ generation from transportation [14]. Given ideal charge transfer and homogeneous transformation, degradation of unit mole of NH_3_ (converting to 1/2 mol of N_2_ with 3 e^−^ transfer) and Total organic carbon (TOC, converting to 1 mol of CO_2_ with 8 e^−^ transfer) would produce 1.5 and 4 mol of H_2_, respectively. Assuming a composition of latrine wastewater to be 20 mM TOC and 10 mM NH_4_^+^, the full mineralization and deammonification would generate a gas mixture with 79% H_2_, 17% CO_2_, and 4% N_2_. This composition would be suitable for further conversion by ignition in internal combustion engine or boiler which would reduce volatilization of toxic chlorinated organic compounds (C_x_H_y_Cl_z_). However, the dependence on the wastewater matrix should be further investigated in a long-term operation study.

## Conclusions

In summary, the NFI/TiO_2_ anode is characterized as Ir-doped spinel NiFe_2_O_4_ in heterojunction with nanoporous anatase TiO_2_ layer, with strong electronic interaction across the junction. The tiny amounts of Ir bring about exceptional intrinsic activity of NFI both for OER (in KOH solutions) and ClER (in NaCl solutions), surpassing the benchmark IrO_2_. The TiO_2_ overlayer enhances the ClER selectivity and durability of NFI during ClER in near-neutral pH. The variation of ECSA, relation of CE_ClER_ with PZC, and the dynamic valency change during *in situ* XANES analysis demonstrated that the upper TiO_2_ serves as the active ClER sites, while the underlying conductive NFI works as the ohmic contact. The charge withdrawing by NFI would facilitate the Cl-chemisorption on charge-deficient TiO_2_. These synergisms allow selective and robust RCS generation on NFI/TiO_2_ architecture, which in turn leads to facile degradation of aqueous pollutants as showcased with stoichiometric NH_4_^+^-to-N_2_ conversion in NH_4_^+^-laden synthetic wastewater. In addition, the alleviated concentrations of dissolved oxygen and RCS can enhance the H_2_ production in single-compartment WEC. The successful operation of scaled-up electrode module for electrolysis of toilet wastewater further substantiated the practical applicability of NFI/TiO_2_. Consequently, NFI/TiO_2_ would be a promising candidate for WEC as an option for on-site wastewater treatment and reuse, with decentralized H_2_ production from nonconventional water sources.

## Supplementary Information

Below is the link to the electronic supplementary material.

Supplementary file1 (DOCX 8538 KB)

## Abbreviations

EOP: Electrochemical oxidation process
ClER: Chlorine evolution reaction
RCS: Reactive chlorine species
WECs: Wastewater electrolysis cells
HER: Hydrogen evolution reaction
BDD: Boron-doped diamond
OER: Oxygen evolution reaction
NF: NiFe_2_O_4_
NFI: Ir-doped NiFe_2_O_4_
FE-SEM: Field emission scanning electron microscope
EDS: Energy-dispersive X-ray spectrometer
XRF: X-ray fluorescence
XRD: X-ray diffraction
XPS: X-ray photoelectron spectroscopy
PAL: Pohang accelerate laboratory
XANES: X-ray absorption near-edge structure
OCV: Open circuit voltage
CI: Current interruption
CV: Cyclic voltammetry
LSV: Linear sweep voltammetry
CDL: Double-layer capacitance
ECSA: Electrochemically active surface area
EIS: Electrochemical impedance spectroscopy
R_s_: Solution resistance
R_f_: Films resistance
R_ct_: Charge transfer resistance
PZC: Potential of zero charge
M-S: Mott–Schottky
DPD: N,N-diethyl-p-phenylenediamine
HHV: Higher heating value
TN: Total nitrogen
COD: Chemical oxygen demand
IC: Ion chromatography
GC-TCD: Gas chromatography with thermal conductivity detector
EEM: Excitation-emission matrix
DOM: Dissolved organic matter
RHE: Reversible hydrogen electrode
CE: Current efficiency
EE: Energy efficiency
TOC: Total organic carbon
