# Supplementary material for: Scalable Ir-Doped NiFe2O4/TiO2 Heterojunction Anode for Decentralized Saline Wastewater Treatment and H2 Production
Source: Nanomicro Lett. 2024 Oct 28;17:51. doi: 10.1007/s40820-024-01542-x (PMC11513779; doi:10.1007/s40820-024-01542-x)
Supplement: Supplementary file 1 — Supplementary file1 (DOCX 8538 KB) [file 40820_2024_1542_MOESM1_ESM.docx]

Supporting Information for

**Scalable Ir-Doped NiFe_2_O_4_/TiO_2_ Heterojunction Anode for Decentralized Saline Wastewater Treatment and H_2_ Production**

Sukhwa Hong^1^, Jiseon Kim^1^, Jaebeom Park^1^, Sunmi Im^1^, Michael R. Hoffmann^2^, and Kangwoo Cho^1,2,3,^*

^1^Division of Environmental Science and Engineering, Pohang University of Science and Technology (POSTECH), Pohang 790−784, Korea

^2^Linde Laboratory, California Institute of Technology, Pasadena, CA 91125, USA

^3^Institute for Convergence Research and Education in Advanced Technology (I-CREATE), Yonsei University International Campus, Incheon 21983, Republic of Korea

*Corresponding author. E-mail: [kwcho1982@postech.ac.kr](mailto:kwcho1982@postech.ac.kr) (Kangwoo Cho)

**Supplementary Tables and Figures**

**Table S1** Energy consumption of various wastewater electrolysis cell using real wastewater samples

| **No.** | **Wastewater** | **Electrode** | **Energy Consumption (kWh g^–^1)** | | | **References** |
| --- | --- | --- | --- | --- | --- | --- |
|  |  |  | **COD** | **TN** | **NH_4_^+^-N** |  |
| 1 | **Toilet wastewater** | **NFI/TiO_2_** | **0.068** | **0.53** | **0.045** | **This work** |
| 2 | Ammonium wastewater | Ti/RuO_2_ mesh | - | 0.160-0.254 | - | [S1] |
| 3 | Landfill leachate (1) | DSA | - | 0.109 | - | [S2] |
| 4 | Landfill leachate (2) |  | - | 0.060 | - |  |
| 5 | Landfill leachate | BDD | 0.114 | - | - | [S3] |
| 6 |  | Pt | 0.095 | - | - |  |
| 7 |  | Pt-IrO_2_ | 0.114 | - | - |  |
| 8 |  | RuO_2_-TiO_2_ | 0.116 | - | - |  |
| 9 |  | RuO_2_-IrO_2_ | 0.110 | - | - |  |
| 10 |  | IrO_2_-Ta_2_O_5_ | 0.112 | - | - |  |
| 11 | Urinary wastewater | BDD | - | 1.32 | - | [S4] |
| 12 | Urinary wastewater | Ti/Ir-SnO_2_ | 0.058 | 0.055 |  | [S5] |
| 13 |  | Ti/IrO_2_ | 0.060 | 0.060 | - |  |
| 14 |  | BDD | 0.085 | 0.092 | - |  |
| 15 | Reverse osmosis concentrates | BDD | 0.059 | - | - | [S6] |
| 16 | Reverse osmosis concentrates | BDD | 0.158-0.203 | - | - | [S7] |
| 17 |  | Ti/IrO_2_-RuO_2_ | 0.048-0.066 | - | - |  |
| 18 |  | Ti/IrO_2_-Ta_2_O_5_ | 0.055-0.211 | - | - |  |
| 19 | Reverse osmosis concentrates | BDD | 0.250 | - | - | [S8] |
| 20 | Textile wastewater | BDD | 0.011 | - | - | [S9] |
| 21 | Textile wastewater | BDD | 0.411-0.865 | - | - | [S10] |
| 22 | Dyeing wastewater | Ti/PbO_2_ | 0.098 | - | - | [S11] |
| 23 | Three brown  diazo dyes | BDD | 0.062 | - | - | [S12] |
| 24 | Industrial wastewater | Ti/PbO_2_ | 0.180 | - | - | [S13] |
| 25 | Tannery effluent | Ti-TiO_2_/IrO_2_/RuO | 0.211 | - | - | [S14] |
| 26 | Slaughterhouse | Ti/Pt mesh | 0.014 | - | - | [S15] |
| 27 | Surfactant | BDD | ~ 1.0 | - | - | [S16] |
| 28 | Aquaculture seawater | Ti/RuO_2_-IrO_2_ | 0.026 | - | 0.054 | [S17] |
| 29 | Aquaculture seawater | Ti/IrO_2_- SnO_2_-Sb_2_O_5_ | - | - | 0.68 | [S18] |
| 30 | Aquaculture seawater | BDD | - | - | 0.041 | [S19] |
| 31 | Aquaculture seawater | Ti/RuO_2_-IrO_2_ | - | - | 0.131 | [S20] |
| 32 | Aquaculture wastewater | Ti/RuO_2_-IrO_2_ | - | - | 0.11 | [S21] |
| 33 | Aquaculture wastewater | Ti/IrO_2_-SnO_2_-Sb_2_O_5_ | - | - | 0.101 | [S22] |
| 35 | Coking wastewater | Ti/RuO_2_-IrO_2_ | - | - | 0.13 | [S23] |
| 36 | Saline industrial wastewater | BDD | - | - | 0.43 | [S24] |


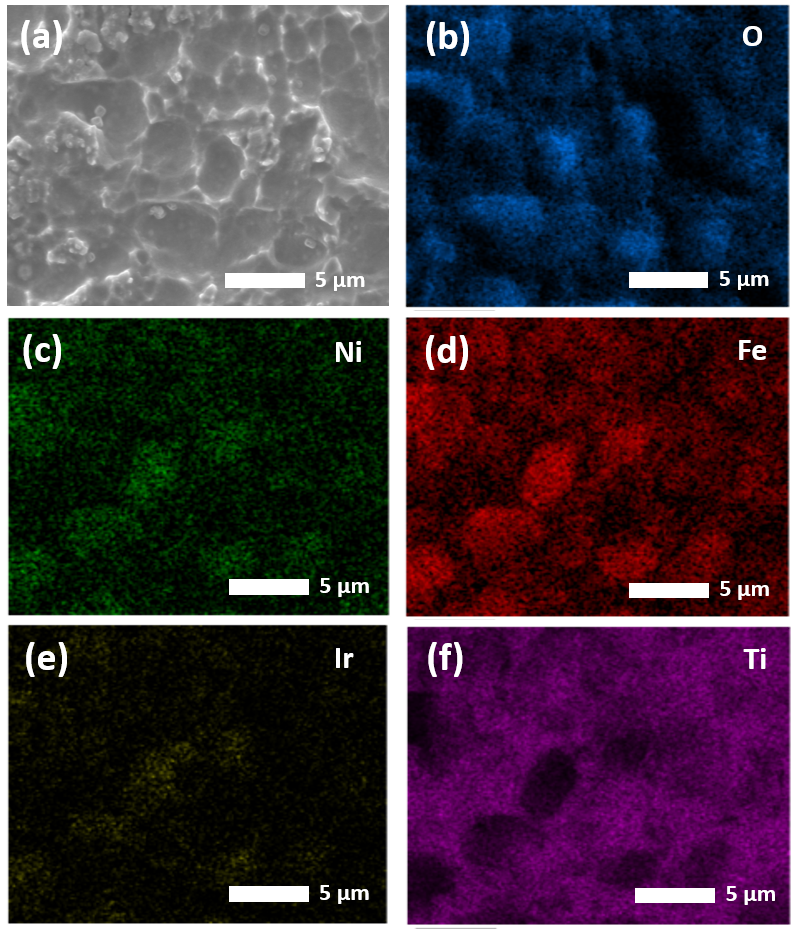


**Fig. S1** (**a**) Scanning electron microscopy (SEM) image of surface view for NFI with energy dispersive spectroscopy (EDS) mapping of (**b**) O (**c**) Ni, (**d**) Fe, (**e**) Ir, and (**f**) Ti

**Fig. S2** X-ray fluorescence spectroscopy (XRF) analysis results of NFI


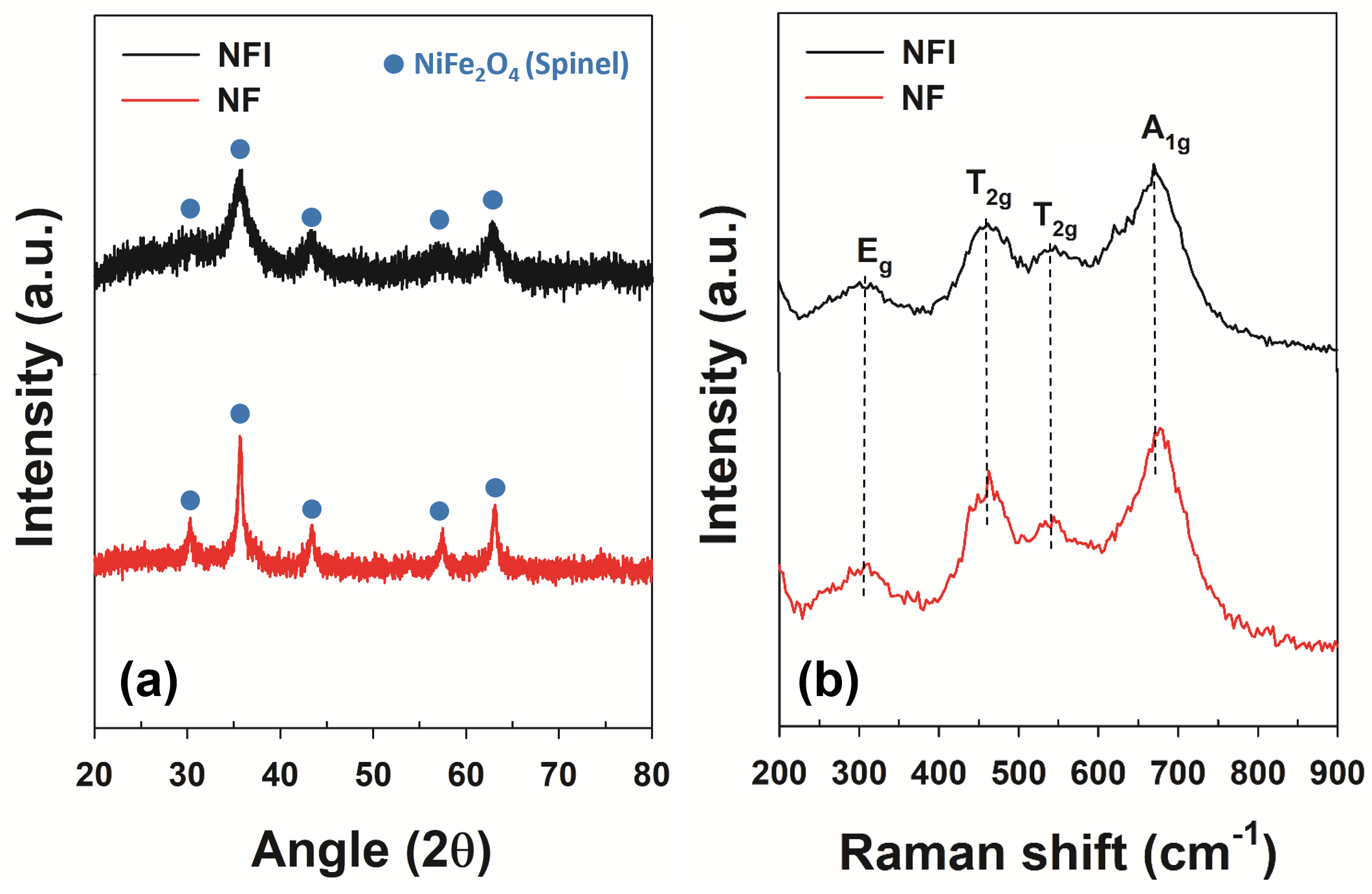


**Fig. S3** (**a**) powder X-ray diffraction (PXRD) profiles for powders of NF and NFI avulsed from Ti substrate with NiFe_2_O_4_ reference (blue circle) and (**b**) Raman spectra for powders of NF and NFI avulsed from Ti substrate with NiFe_2_O_4_ reference (A1g, T2g, and Eg)


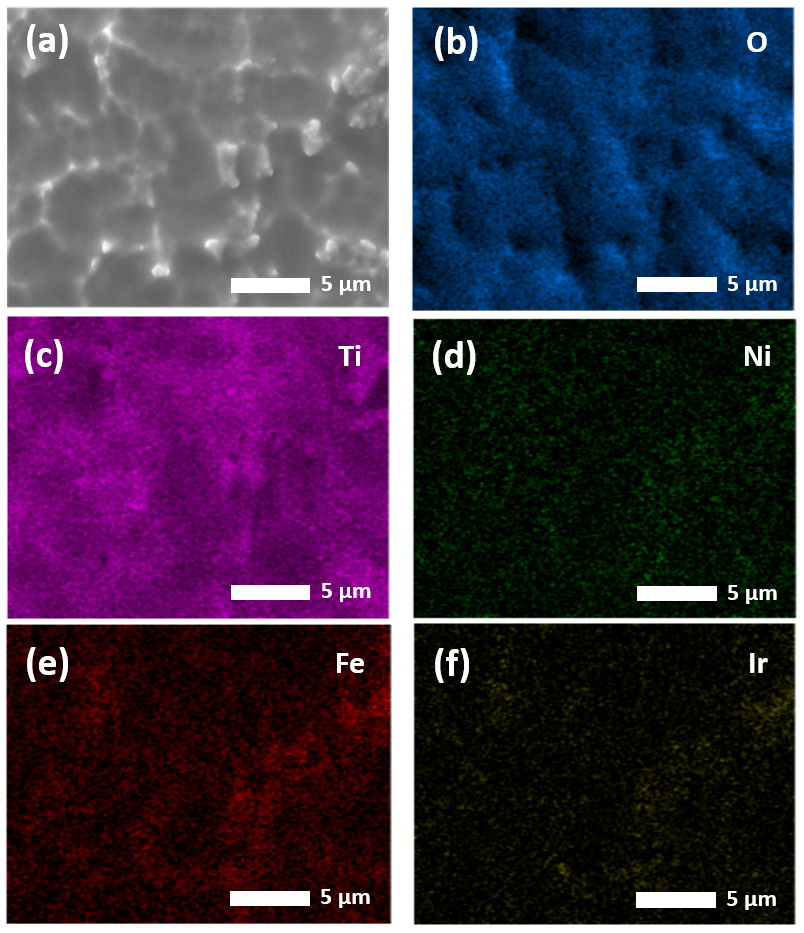


**Fig. S4** (**a**) Scanning electron microscopy (SEM) image of surface view for NFI/TiO_2_ with energy dispersive spectroscopy (EDS) mapping of (**b**) O (**c**) Ti, (**d**) Ni, (**e**) Fe, and (**f**) Ir

**Fig. S5** Glow Discharge Spectrometer (GDS) analysis of NFI/TiO_2_


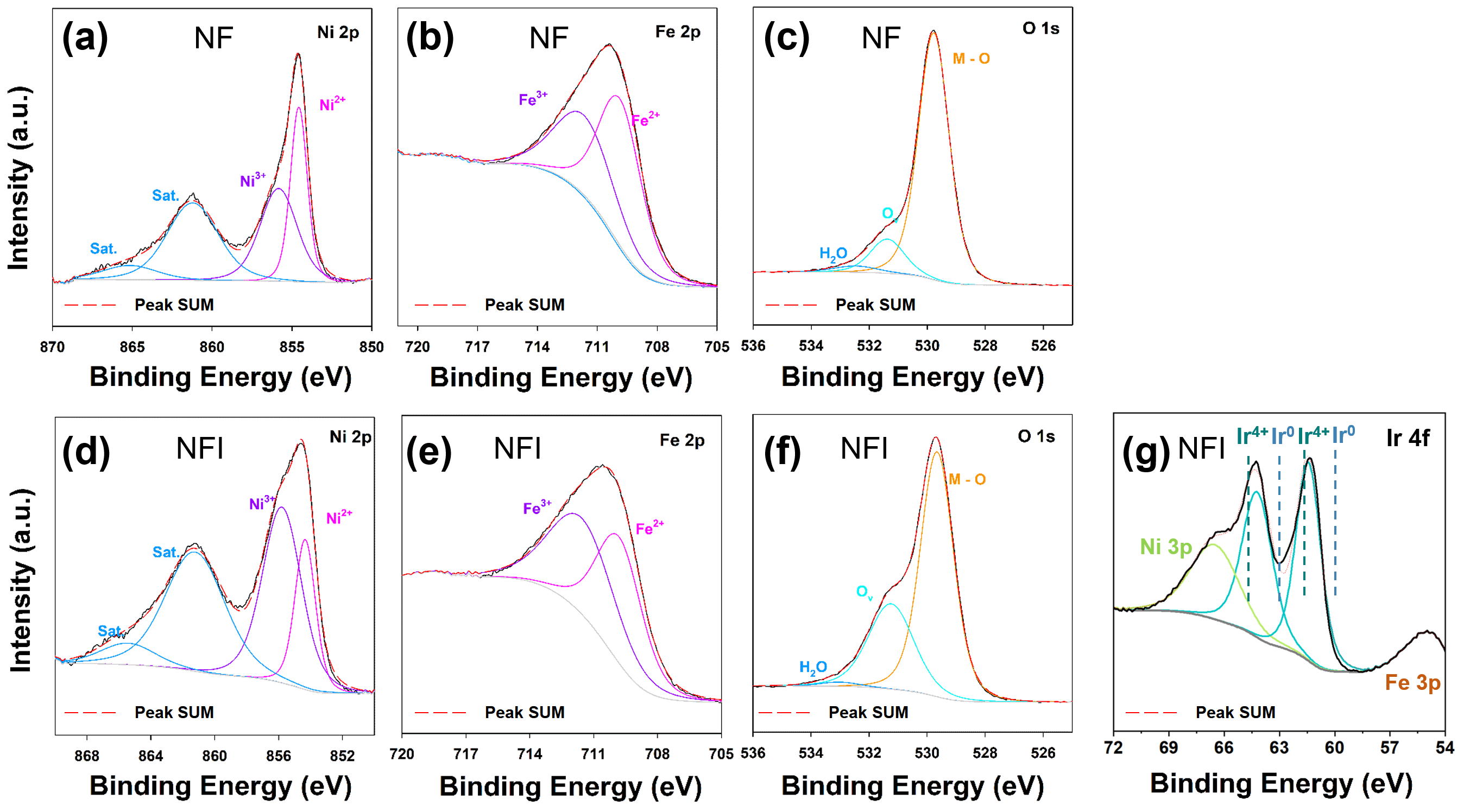


**Fig. S6** Deconvolution data of (**a**) Ni 2p, (**b**) Fe, and (**c**) O 1s, 2p from X-ray photoelectron spectroscopy (XPS) spectra of NF. Deconvolution data of (**d**) Ni 2p, (**e**) Fe 2p (**f**) O 1s, and (**g**) Ir 4f from X-ray photoelectron spectroscopy (XPS) spectra of NFI


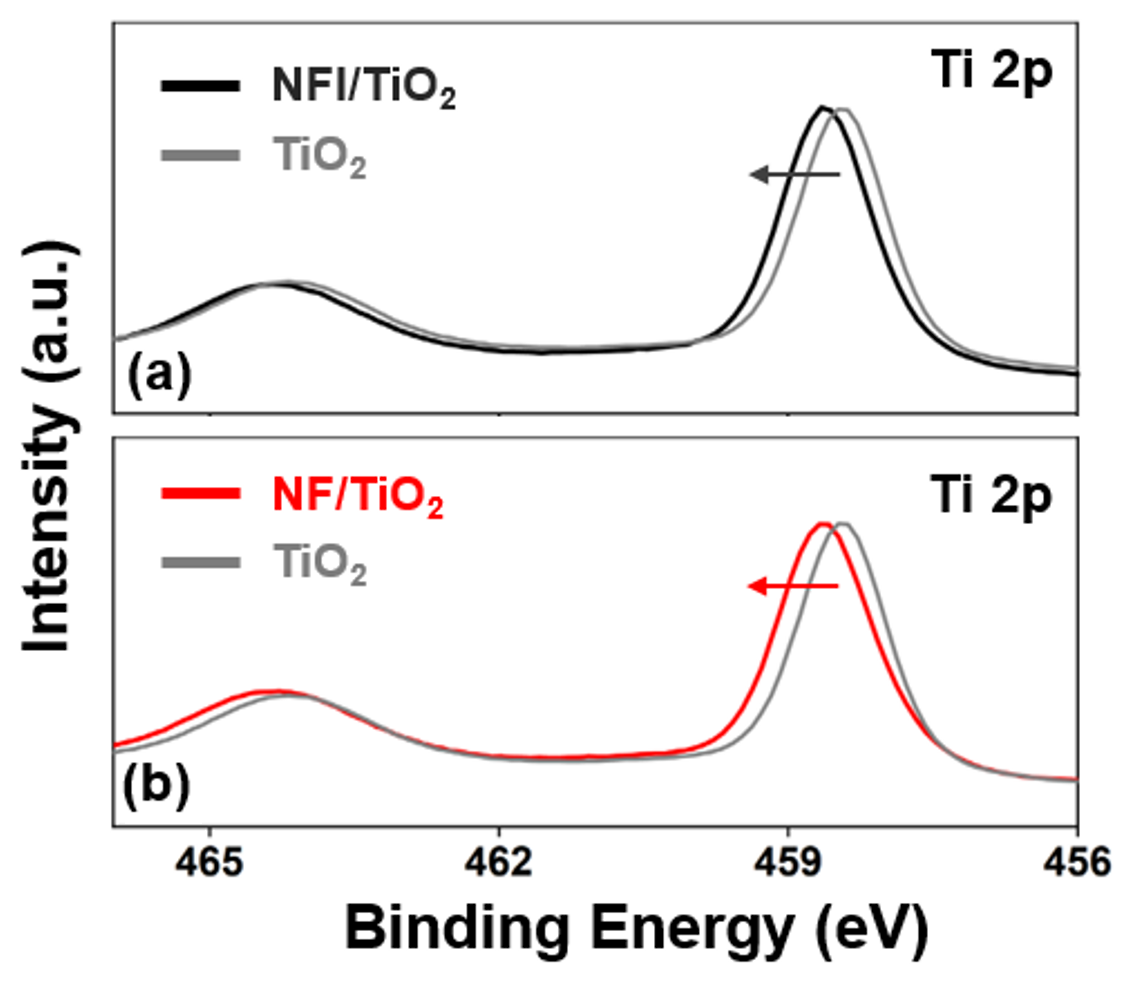


**Fig. S7** Ti 2p peak comparison in X-ray photoelectron spectroscopy (XPS) spectra for NF/TiO_2_ and NFI/TiO_2_ with TiO_2_ on Ti substrate


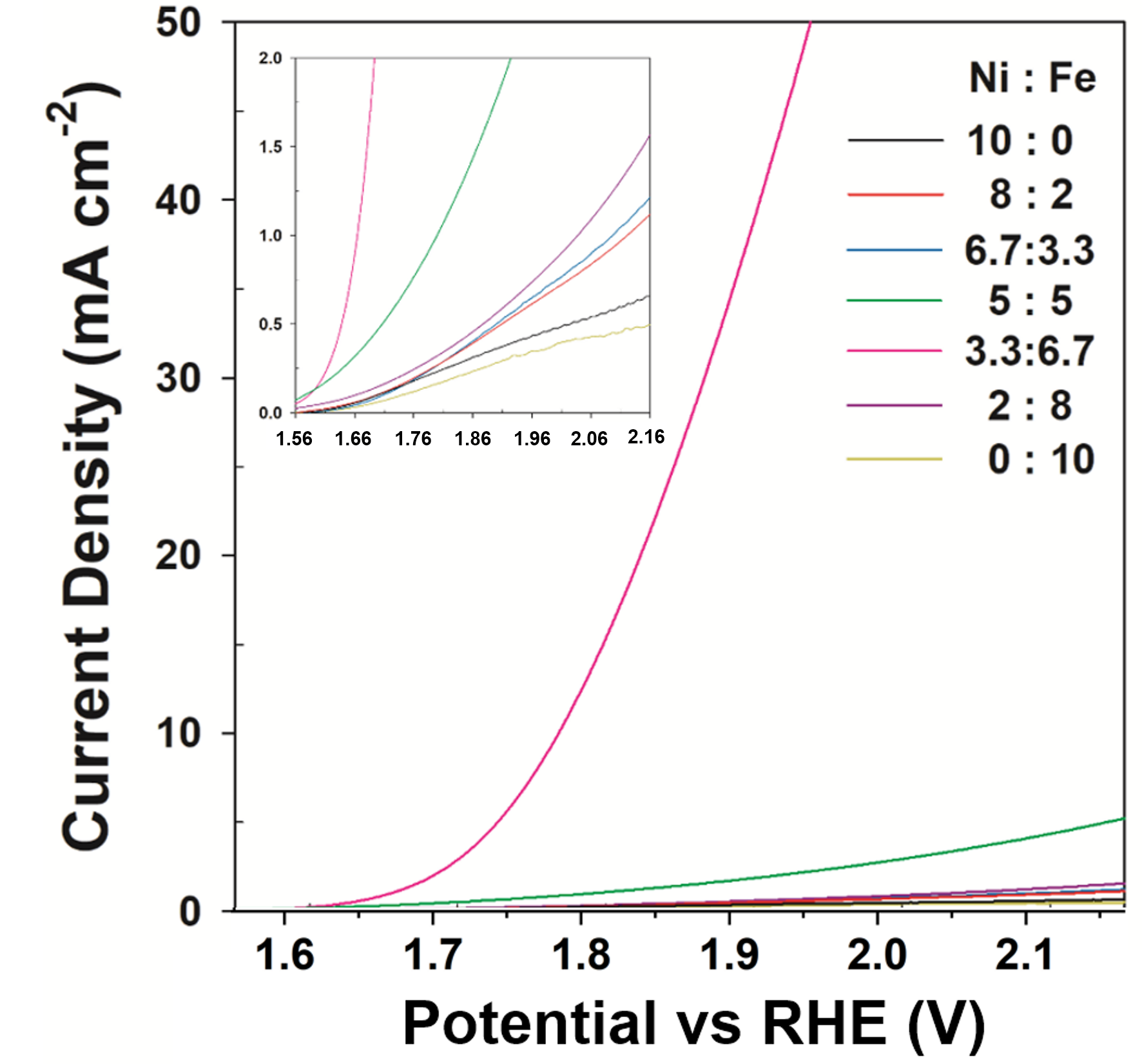


**Fig. S8** Linear sweep voltammograms of the nickel and iron oxides synthesized on Ti substrate from nitrate salt precursor using sol-gel and thermal decomposition method (Cathode: Pt, reference: Hg/HgO, geometric surface area: 2 × 1 cm^2^, and scan rate: 20 mV s^−1^)

**
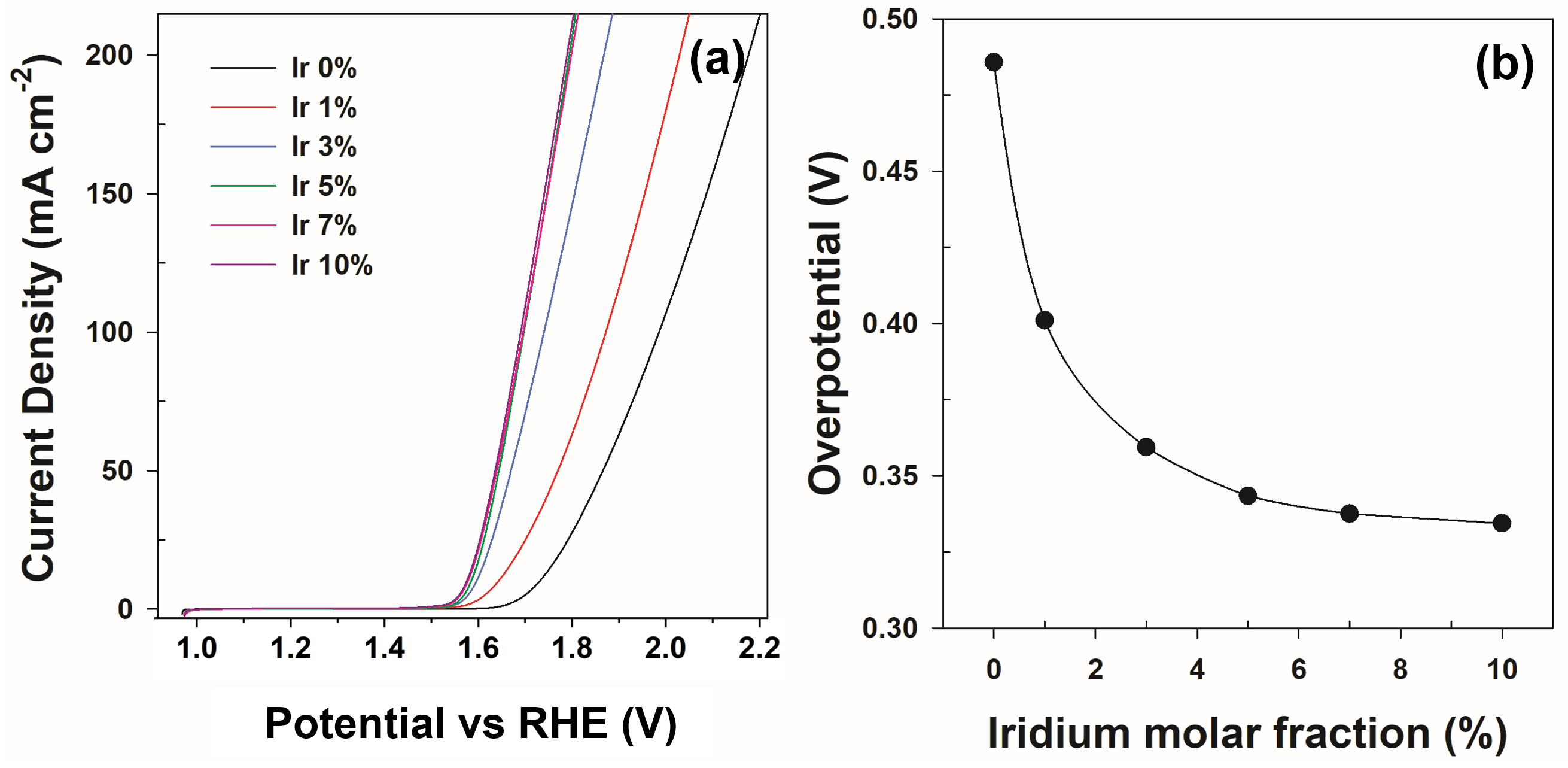
 Fig. S9** (**a**) Linear sweep voltammograms and (**b**) overpotential at 10 mA cm^-2^ of the Ir doped NiFe_2_O_4_ synthesized on Ti substrate by mixing nickel ferrite and iridium precursor as molar ratio (Ir = 0, 1, 3, 5, 7, and 10%) and proceeding sol-gel and thermal decomposition method (Cathode: Pt, reference: Hg/HgO, geometric surface area: 2 × 1 cm^2^, and scan rate: 20 mV s^−1^)


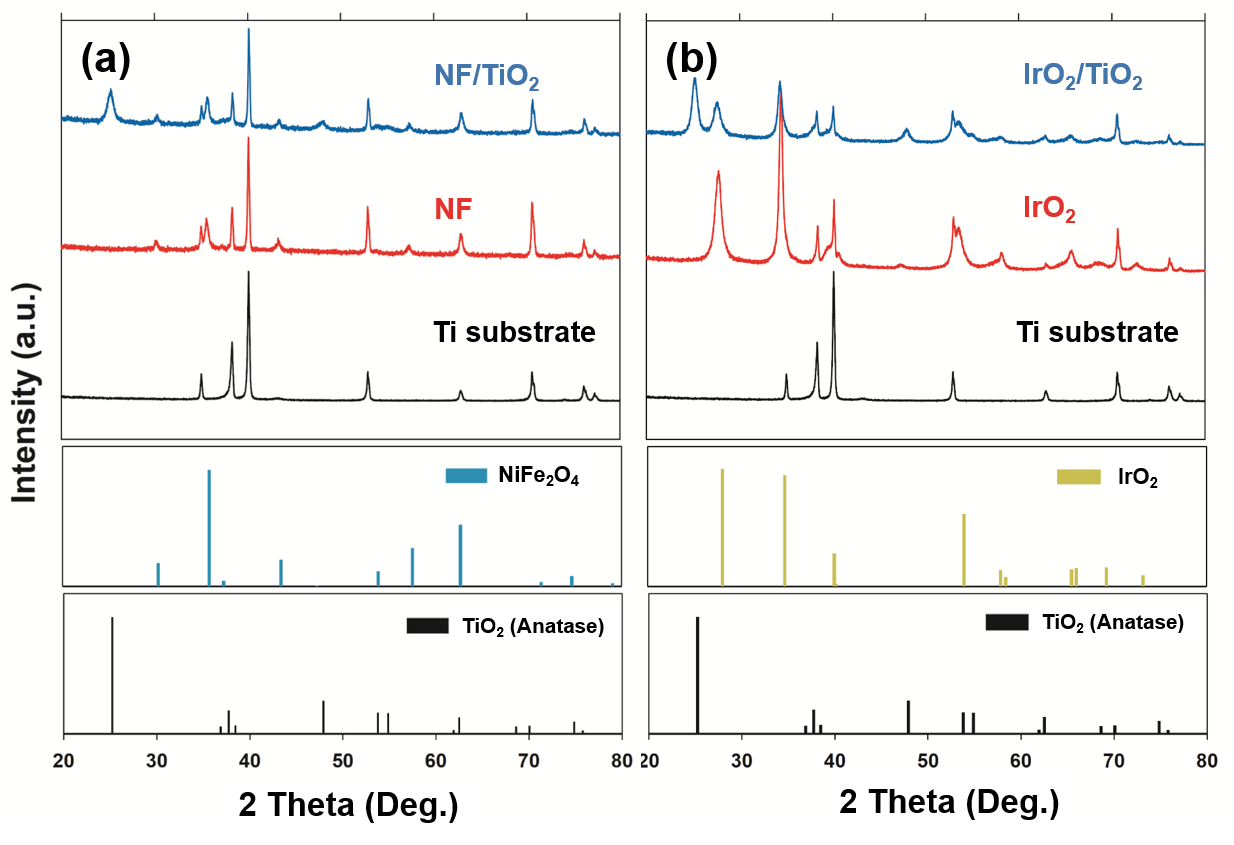


**Fig. S10** (**a**) X-ray diffraction patterns (XRD) of Ti substrate, NF, and NF/TiO_2_ heterojunction anodes referenced with NiFe_2_O_4_ and TiO_2_ (Anatase) and (**b**) Ti substrate, IrO_2_, and IrO_2_/TiO_2_ heterojunction anodes referenced with IrO_2_ and TiO_2_ (Anatase)


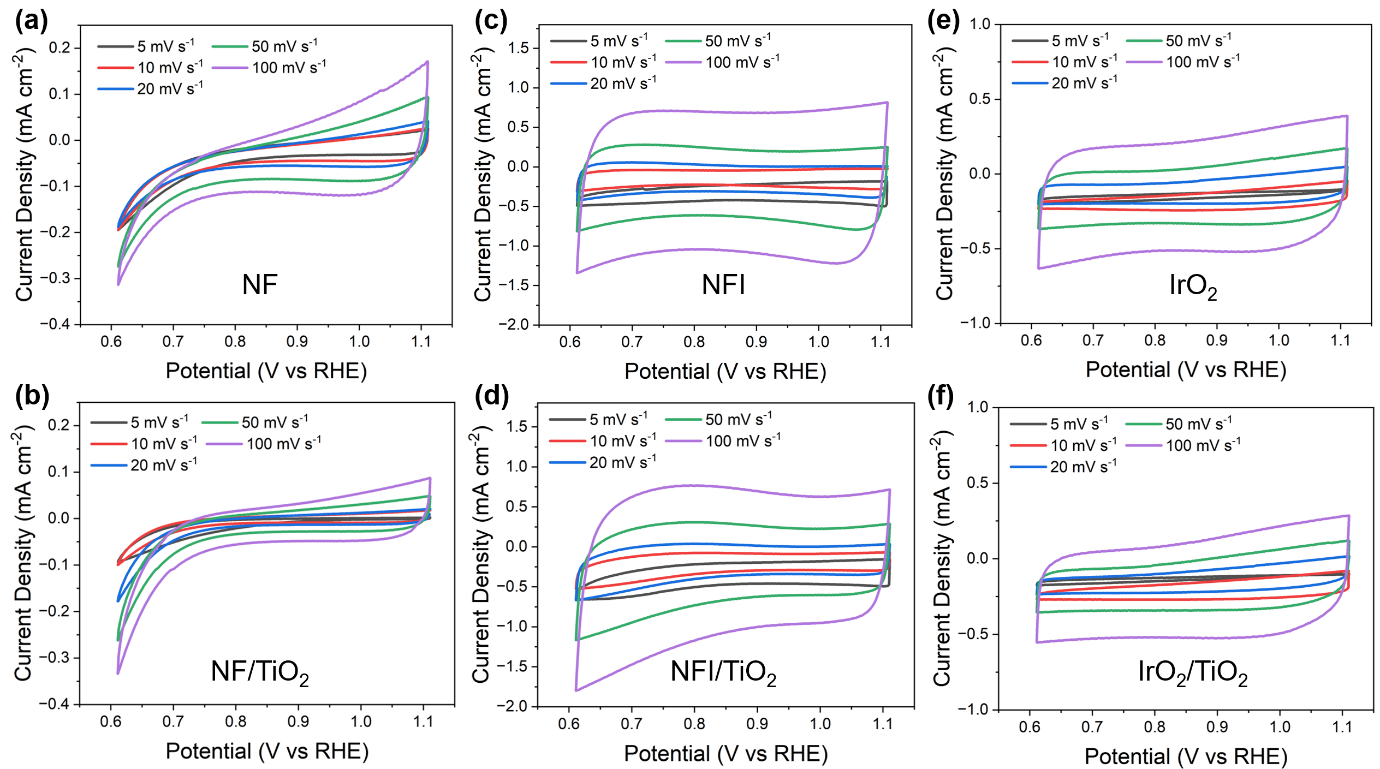
**Fig. S11** Cyclic voltammograms for double layer capacitance measurement of (**a**) NF, (**b**) NF/TiO_2_, (**c**) NFI, (**d**) NFI/TiO_2_, (**e**) IrO_2_, and (**f**) IrO_2_/TiO_2_ with scan range of 0−0.5 V vs Ag/AgCl in 100 mM NaCl solution (Cathode: Pt, reference: Ag/AgCl, geometric surface area: 2 × 1 cm^2^, scan rates = 5, 10, 20, 50, and 100 mV s^−1^)

**
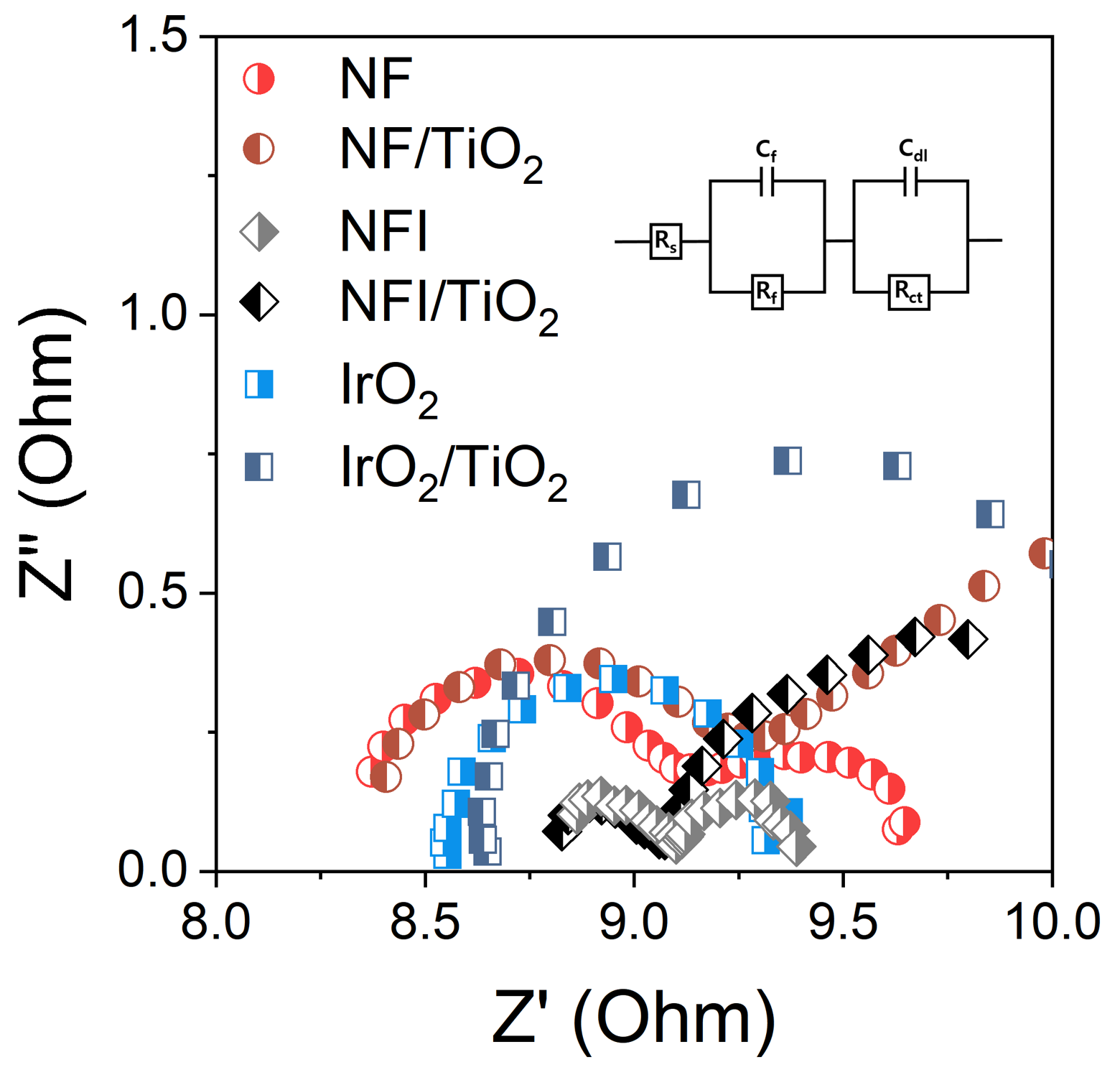
**

**Fig. S12** Nyquist plots from electrochemical impedance spectroscopy (EIS) results (scan frequency: 100 kHz − 10 Hz, 1.9 V RHE) for NFI, NF, and IrO_2_ electrocatalysts in 100 mM NaCl

**
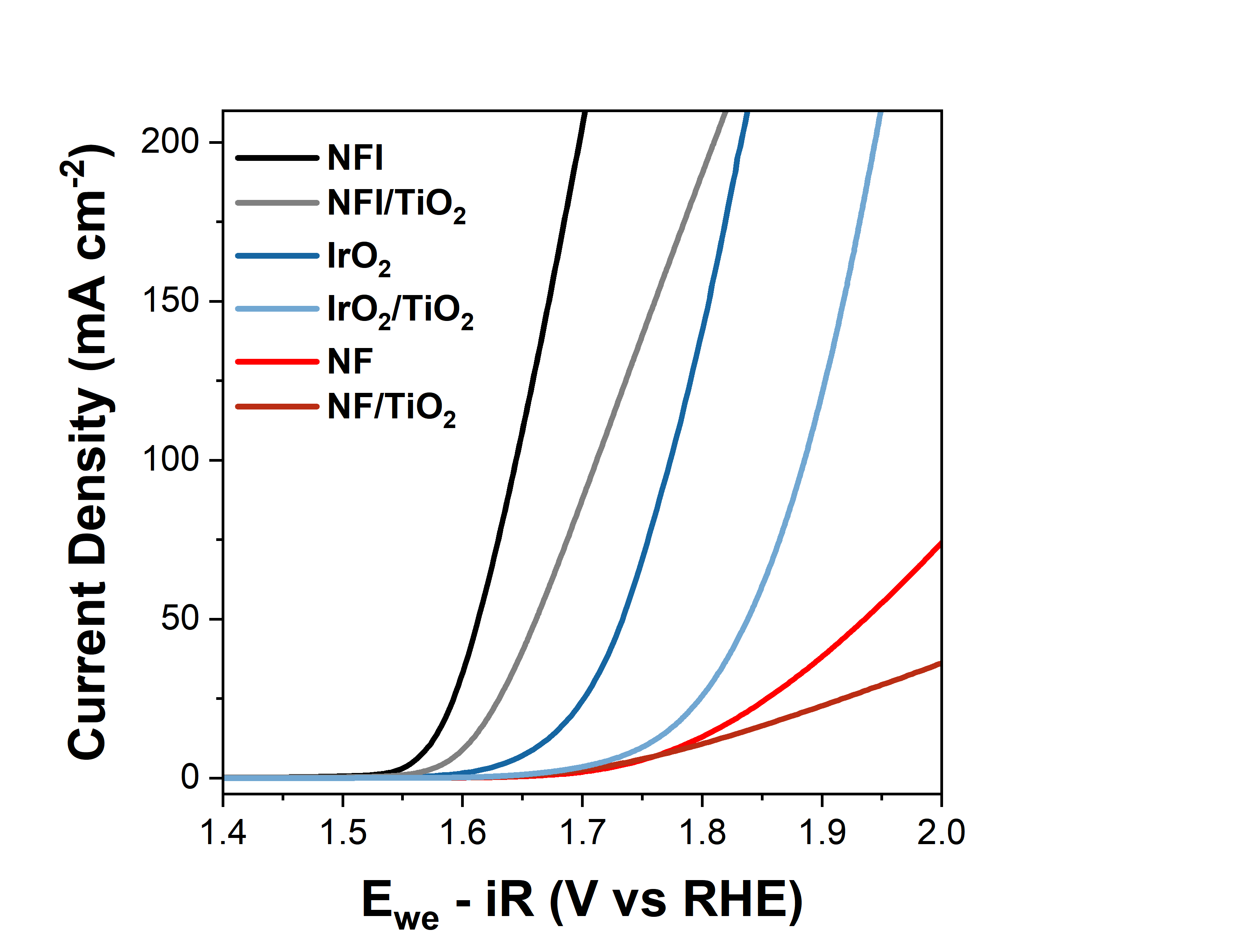
**

**Fig. S13** iR compensated linear sweep voltammograms for NFI, NFI/TiO_2_, NF, NF/TiO_2_, IrO_2_, and IrO_2_/TiO_2_ heterojunction electrodes in 1 M KOH


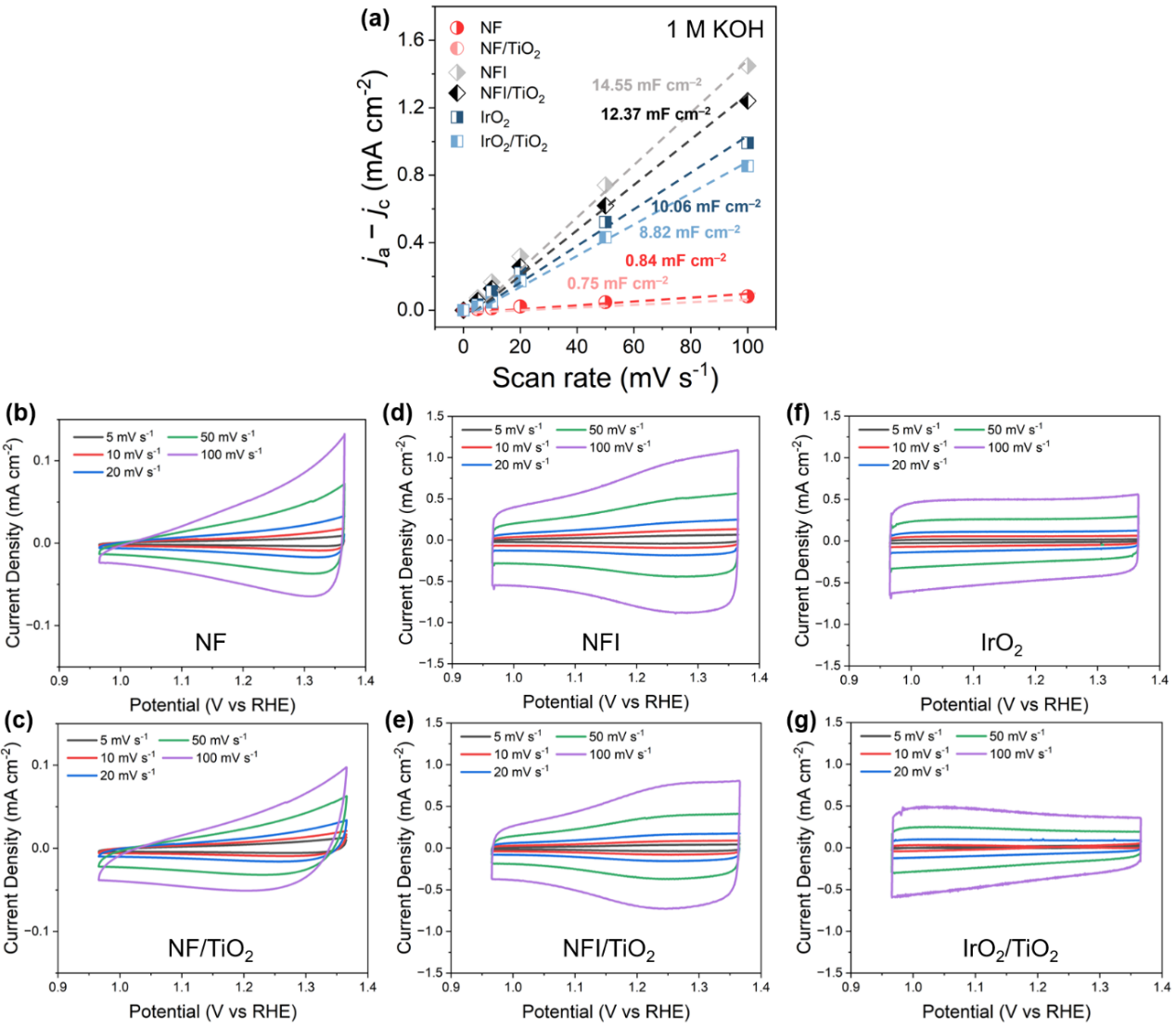


**Fig. S14** (**a**) Capacitive *j*_a_-*j*_c_ vs scan rate from cyclic voltammograms for NFI, NFI/TiO_2_, NF, NF/TiO, IrO_2_, and IrO_2_/TiO_2_ electrocatalysts in 1 M KOH. Cyclic voltammograms for double layer capacitance measurement of (**b**) NF, (**c**) NF/TiO_2_, (**d**) NFI, (**e**) NFI/TiO_2_, (**f**) IrO_2_, and (**g**) IrO_2_/TiO_2_ with scan range of 0−0.4 V vs Hg/HgO in 1 M KOH solution (Cathode: Pt, reference: Hg/HgO, geometric surface area: 2 × 1 cm^2^, scan rates = 5, 10, 20, 50, and 100 mV s^−1^)


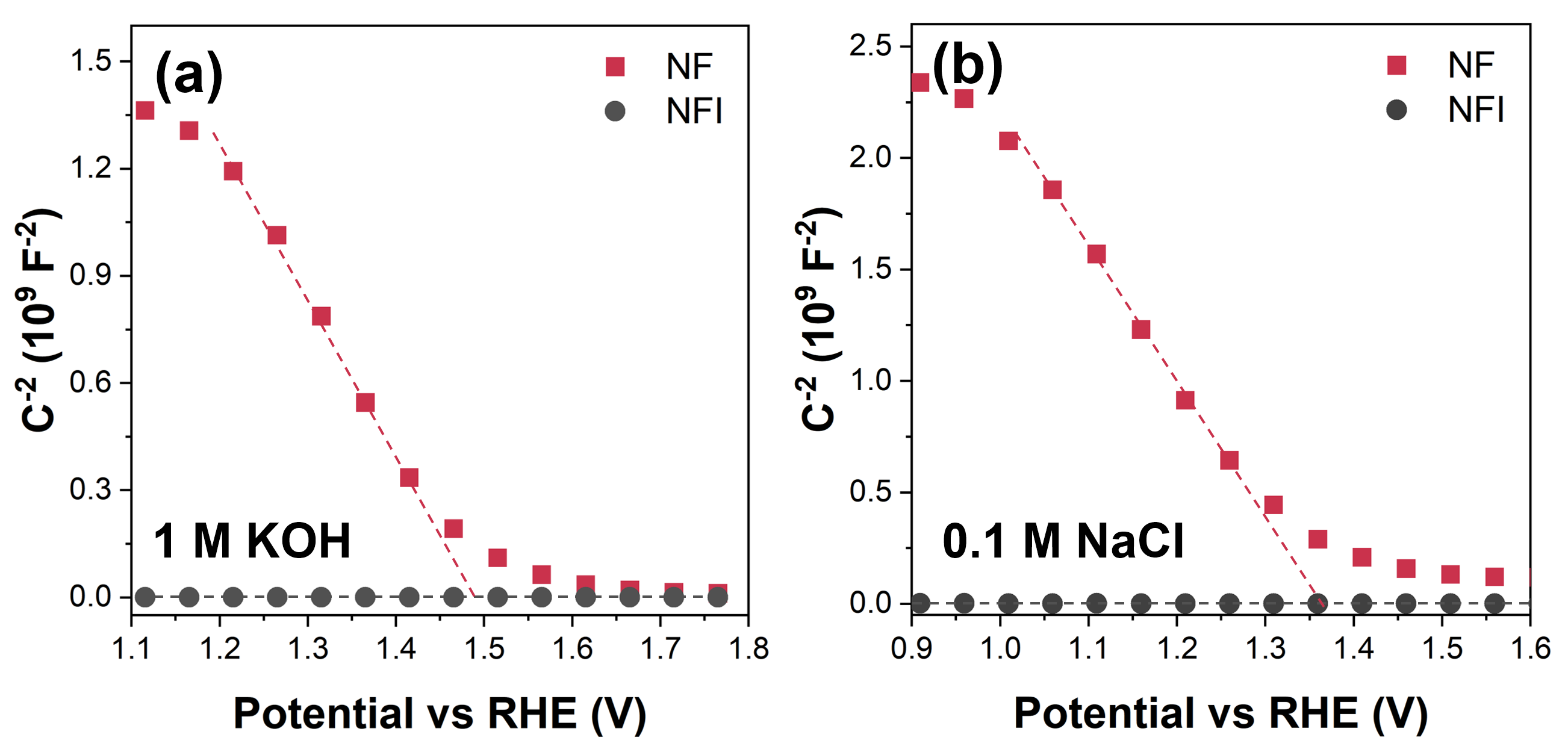


**Fig. S15** Mott-Schottky plots in electrochemical impedance spectroscopy. The Mott-Schottky plots were measured (**a**) in aqueous 1 M KOH with a frequency of 3.2 kHz with the potential range of 0.1 to 0.9 V vs Hg/HgO and (**b**) in 0.1 M NaCl with the potential range of 0.2 to 1.0 V vs Ag/AgCl


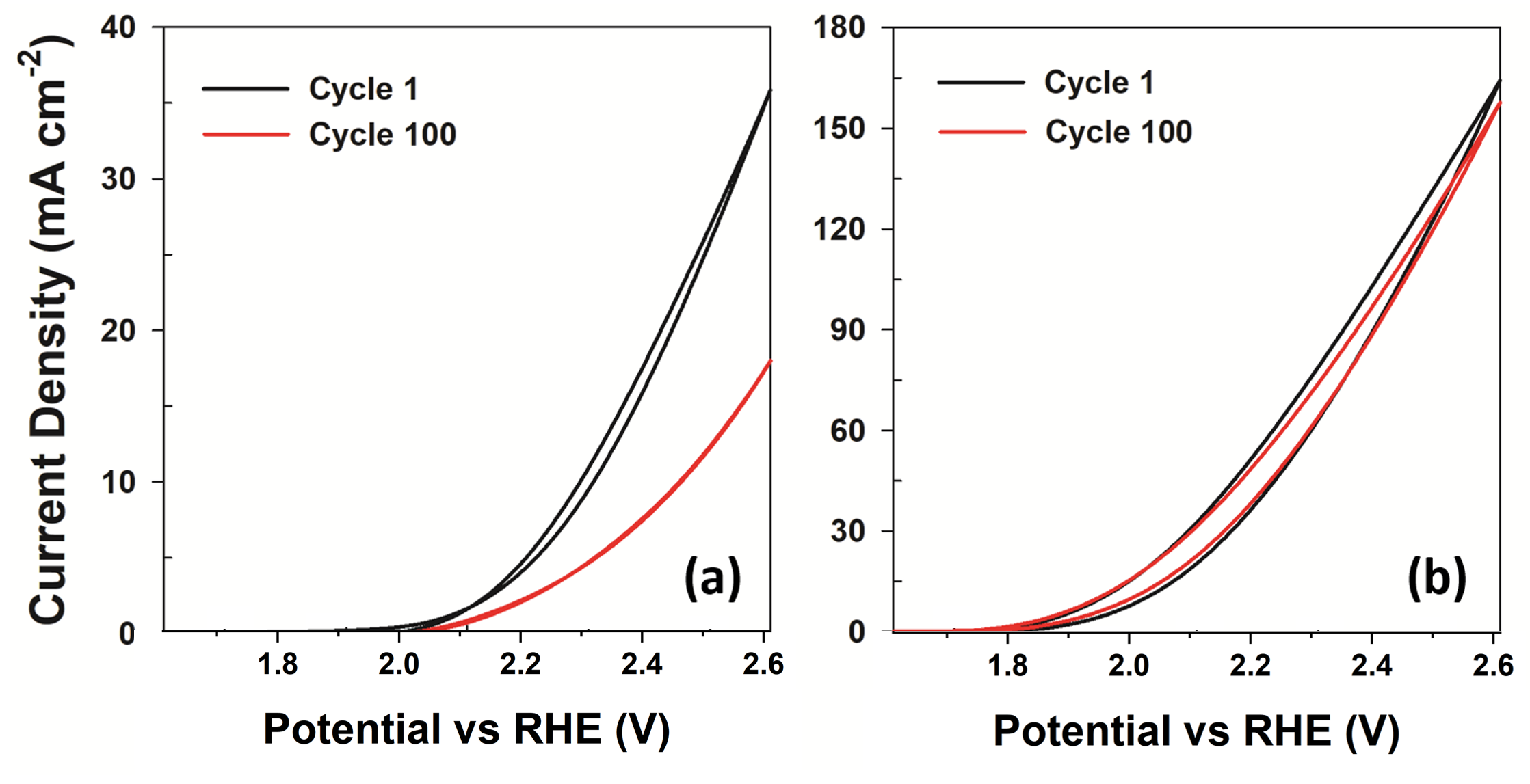


**Fig. S16** Cyclic voltammograms of 100 cycles for stability test of (a) NF/TiO_2_ and (b) NFI/TiO_2_ in 0.5 M NaClO_4_ solution with scan rates of 20 mV s^−1^ (Cathode: Pt, reference: Ag/AgCl, and geometric surface area: 2 × 1 cm^2^)


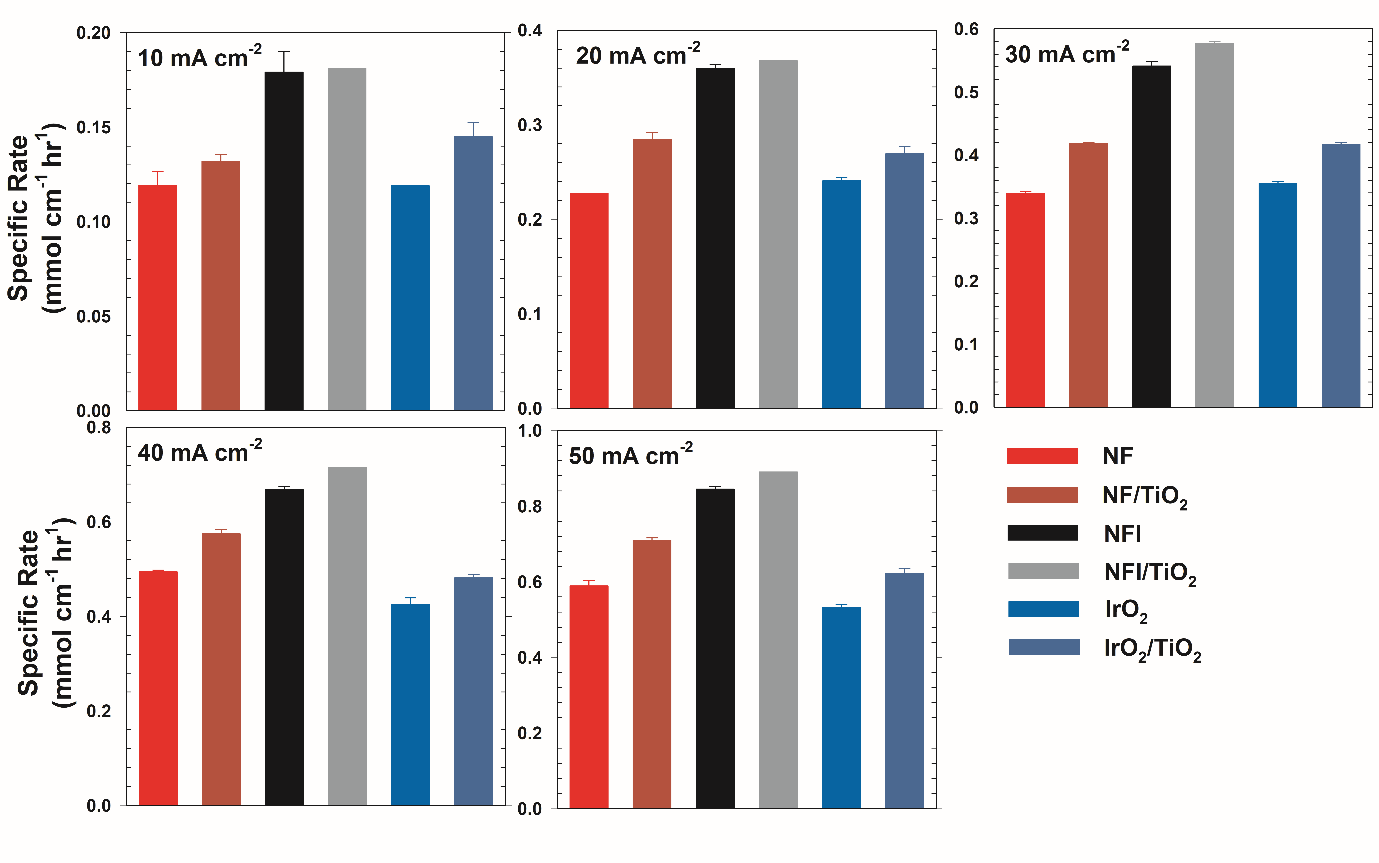


**Fig. S17** Specific rate for ClER (SR_ClER_) of NF, NF/TiO_2_, NFI, NFI/TiO_2_, IrO_2_, and IrO_2_/TiO_2_ at all galvanostatic conditions in 100 mM NaCl solutions (Cathode: Pt, reference: Ag/AgCl, geometric surface area: 2 × 1 cm^2^, and applied anodic current density: 10, 20, 30, 40, and 50 mA cm^-2^)

**
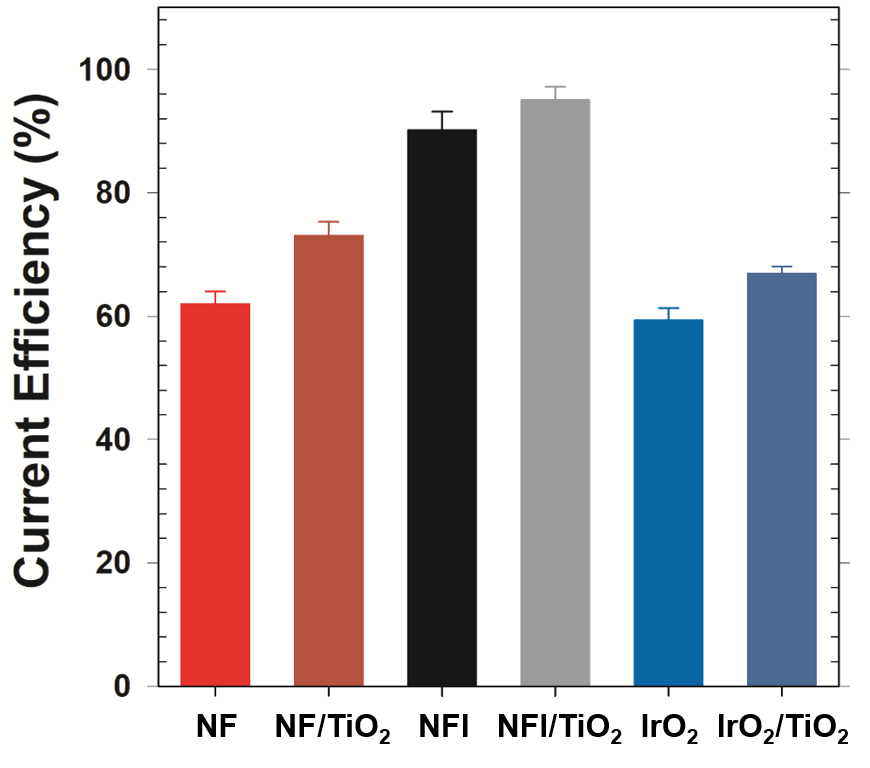
**

**Fig. S18** Average of current efficiency for RCS generation (CE_RCS_) of NF, NF/TiO_2_, NFI, NFI/TiO_2_, IrO_2_, and IrO_2_/TiO_2_ at all galvanostatic conditions in 100 mM NaCl solutions (Cathode: Pt, reference: Ag/AgCl, geometric surface area: 2 × 1 cm^2^, and applied anodic current density: 10, 20, 30, 40, and 50 mA cm^-2^)

**
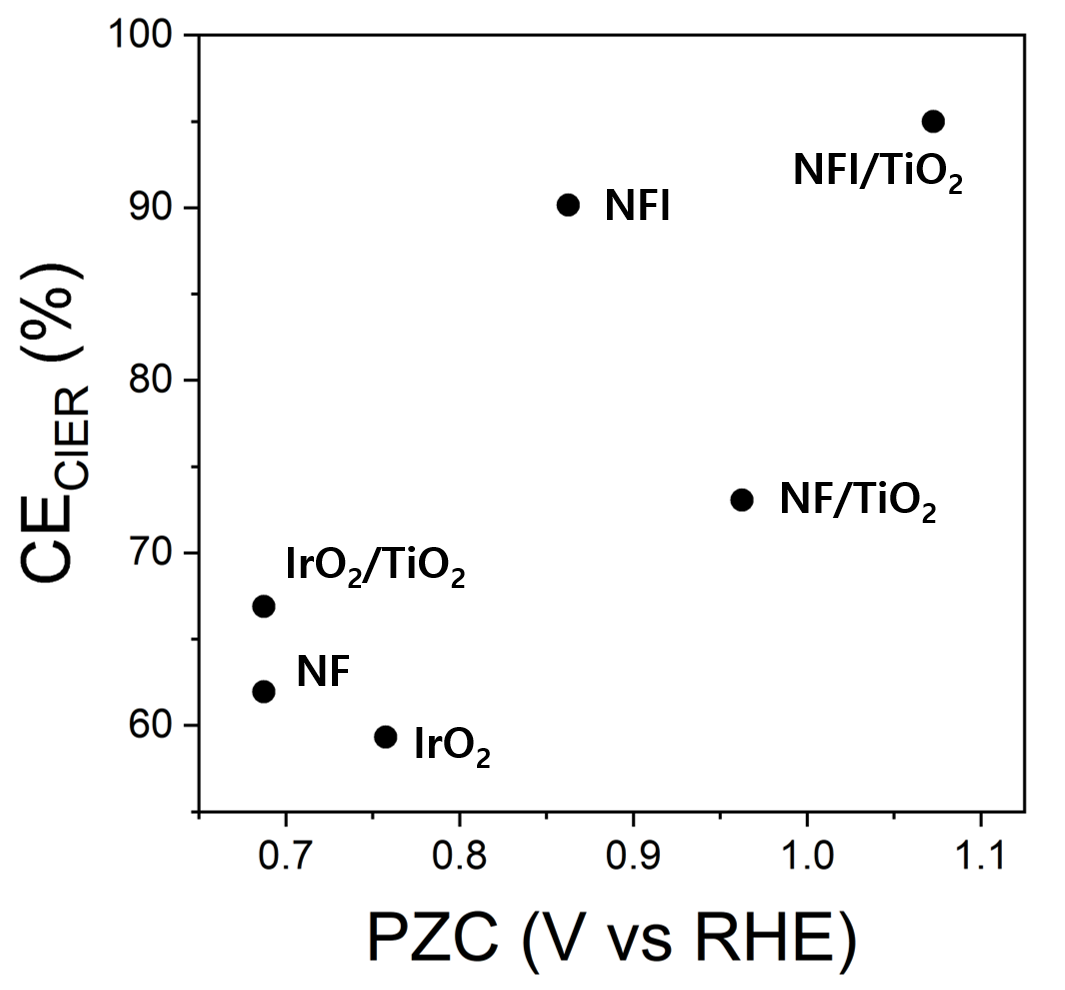
**

**Fig. S19** Correlations of PZC with CE_ClER_ of NF, NF/TiO_2_, NFI, NFI/TiO_2_, IrO_2_, and IrO_2_/TiO_2_

**
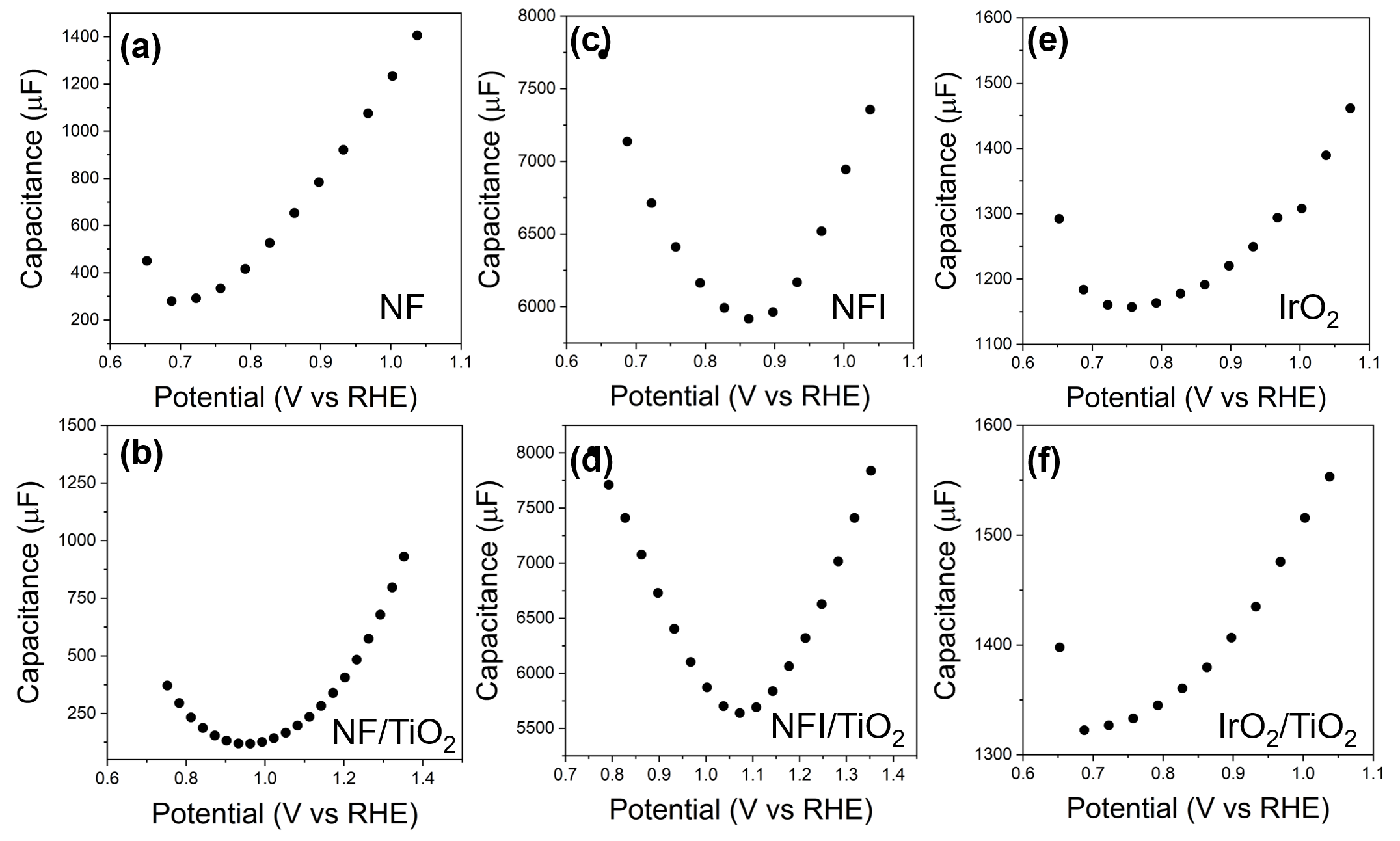
**

**Fig. S20** Evolutions of capacitance as functions of applied anodic potential (E) for (**a**) NF, (**b**) NF/TiO_2_, (**c**) NFI, (**d**) NFI/TiO_2_, (**e**) IrO_2_, and (**f**) IrO_2_/TiO_2_ to estimate the potential of zero charge (electrolyte = 0.1 M NaCl, frequency = 150 mHz, sinus amplitude = 5 mV)


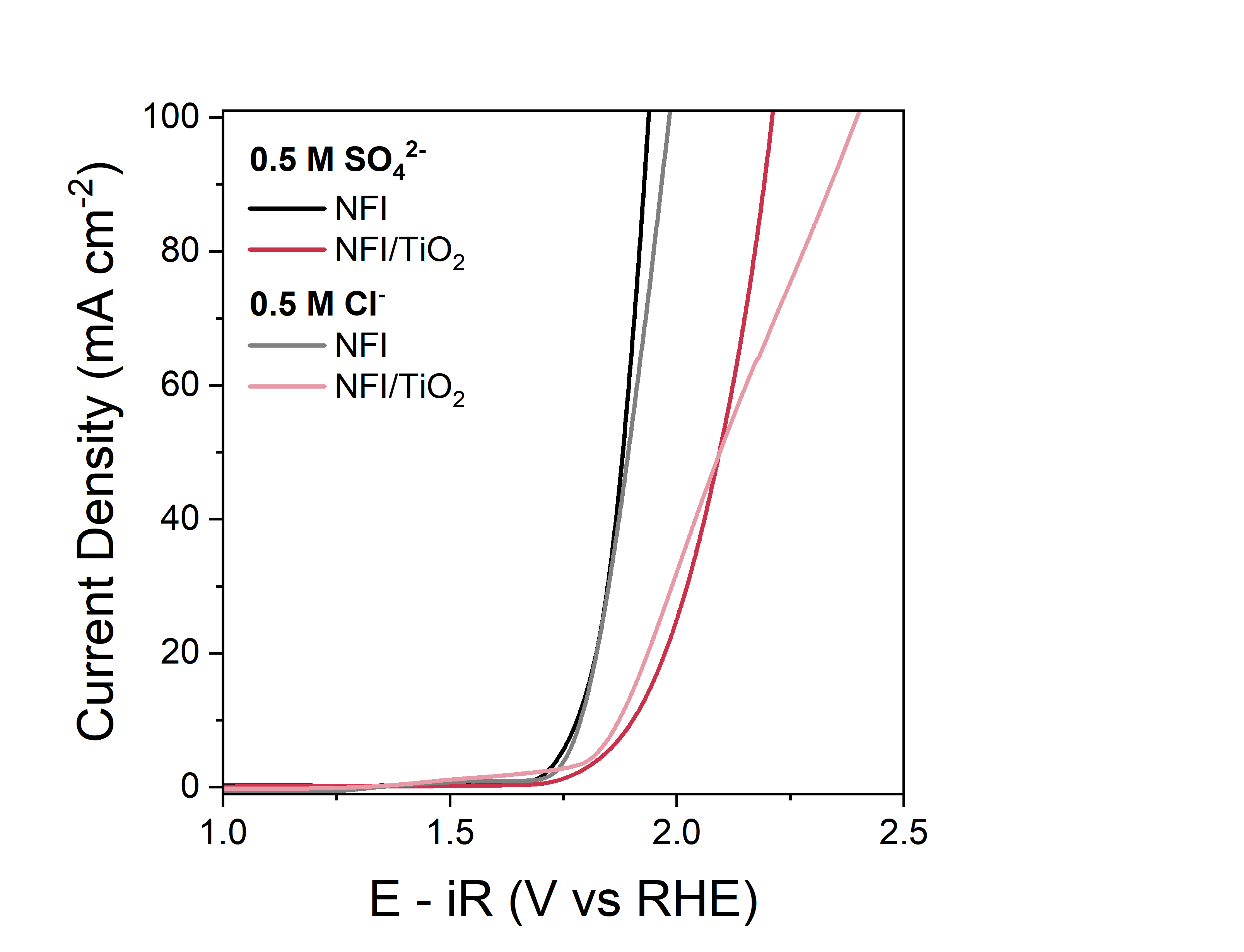


**Fig. S21** *iR*-compensated LSVs of NFI and NFI/TiO_2_ in 0.5 M Na_2_SO_4_ (for exclusive OER) or 0.5 M NaCl (for predominant ClER)


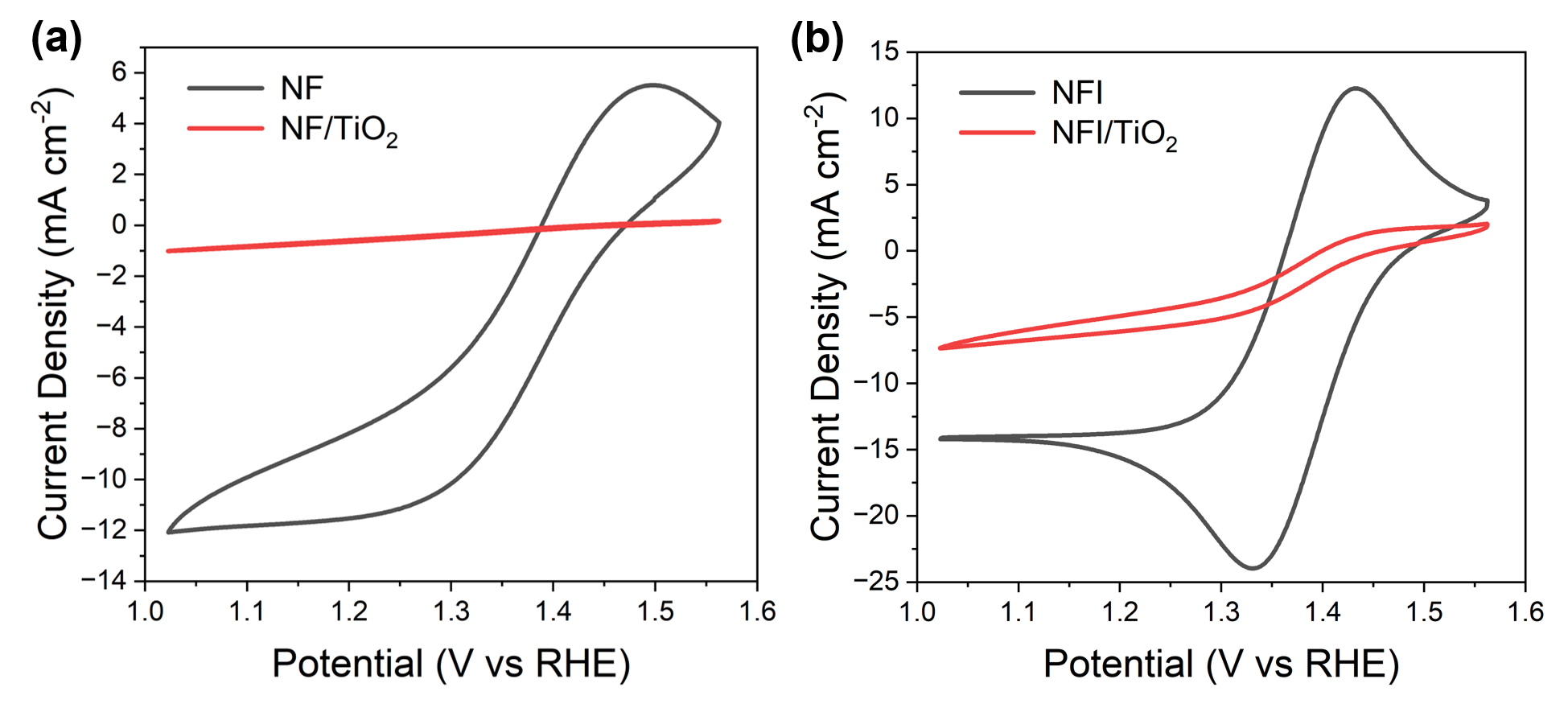


**Fig. S22** Cyclic voltammograms of (**a**) NF and NF/TiO_2_ and (**b**) NFI and NFI/TiO_2_ in 1 M KOH + 0.1 M K_3_Fe(CN)_6_ solution

**Fig. S23** Concentration for chloride and nitrite during the electrolysis process for NFI/TiO_2_, BDD, and IrO_2_/TiO_2_ under 30 mA cm^-2^ for 3 h (Cathode: Pt, reference: Ag/AgCl, geometric surface area: 2 × 1 cm^2^, [Ammonium]_0_ = 25 mM, [Cl^-^]_0_ = 100 mM)


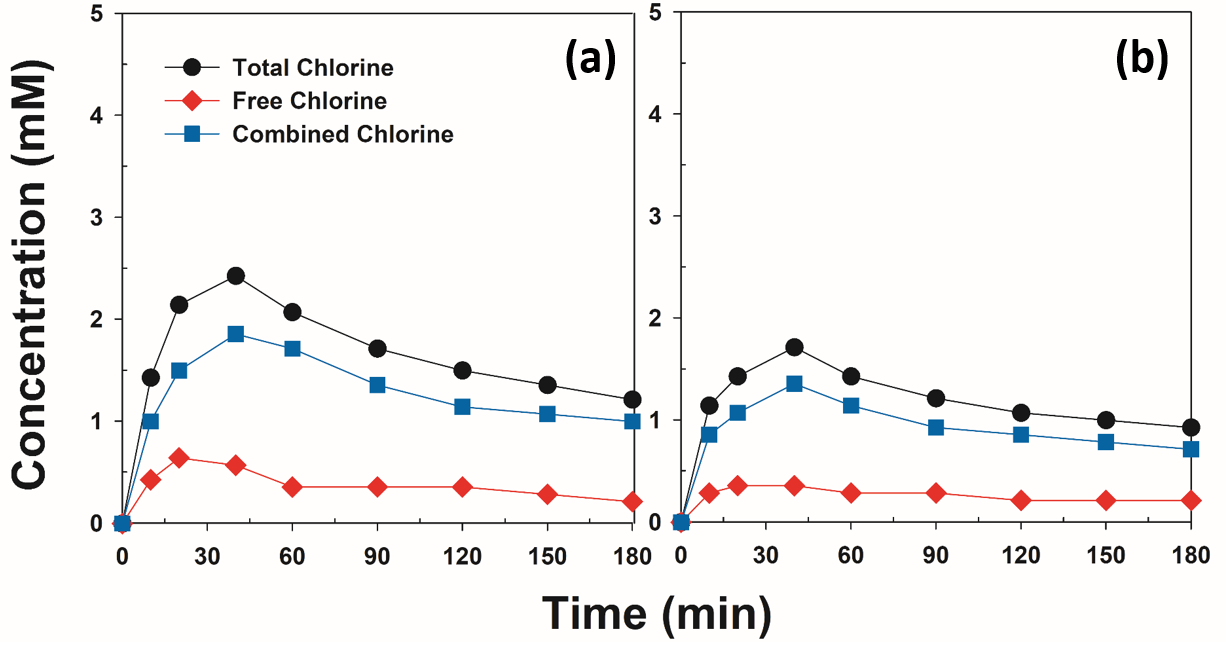


**Fig. S24** Time profiles of the concentration for total chlorine, free chlorine, and combined chlorine of (**a**) BDD and (**b**) IrO_2_/TiO_2_ under 30 mA cm^-2^ for 3h (Cathode: Pt, reference: Ag/AgCl, geometric surface area: 2 × 1 cm^2^, [Ammonium]_0_ = 25 mM, and [Cl^-^]_0_ = 100 mM)


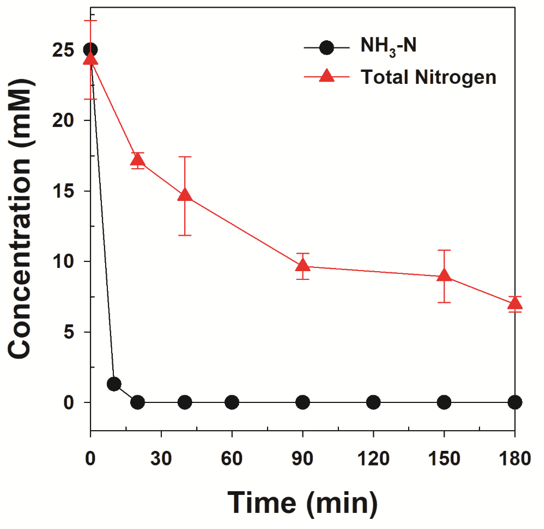


**Fig. S25** Time profiles of the concentration for total nitrogen and ammonium N of chemical experiment stirred for 3 hours ([Ammonium]_0_ = 25 mM and [OCl^-^]_0_ = 100 mM)


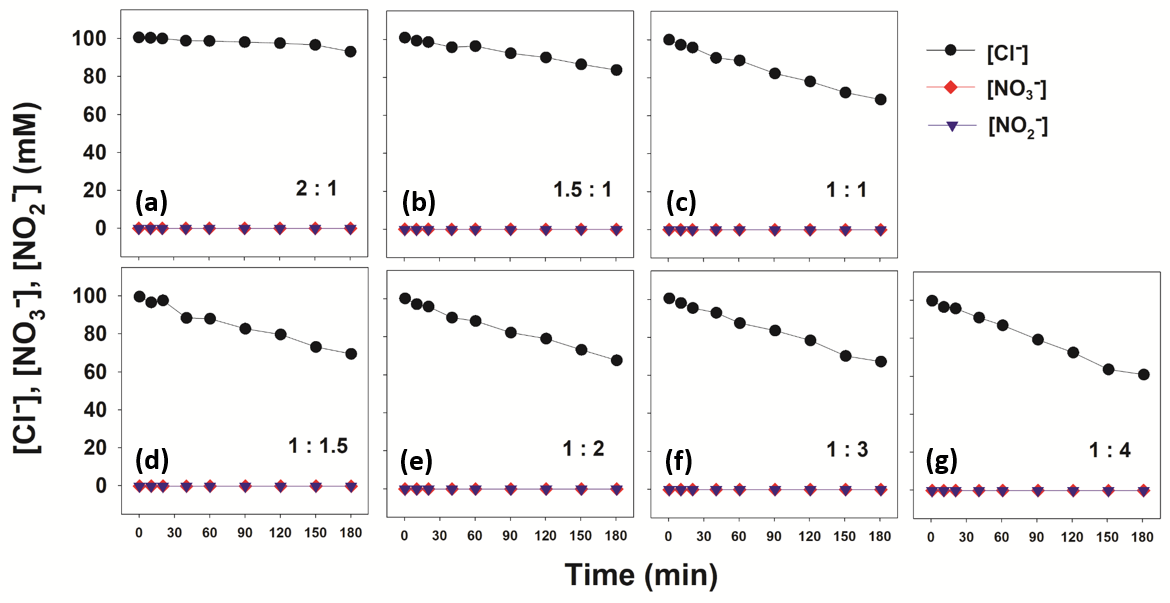


**Fig. S26** Time profiles of concentration for chloride, nitrate, and nitrite of NFI/TiO_2_ in initial 100 mM Cl^-^ with various initial ammonia concentration (NH_4_^+^:Cl^−^ molar ratio, 2:1, 1.5:1, 1:1, 1:1.5, 1:2, 1:3, and 1:4) under 30 mA cm^-2^ for 3h (Cathode: Pt, reference: Ag/AgCl, and geometric surface area: 2 × 1 cm^2^)

**Fig. S27** Time profiles of the concentration for (**a**) total chlorine and (**b**) free chlorine of NFI/TiO_2_ in initial 100 mM Cl^−^ with various initial ammonia concentration (NH_4_^+^:Cl^−^ molar ratio, 2:1, 1.5:1, 1:1, 1:1.5, 1:2, and 1:3) under 30 mA cm^−2^ for 3h (Cathode: Pt, reference: Ag/AgCl, geometric surface area: 2 × 1 cm^2^)


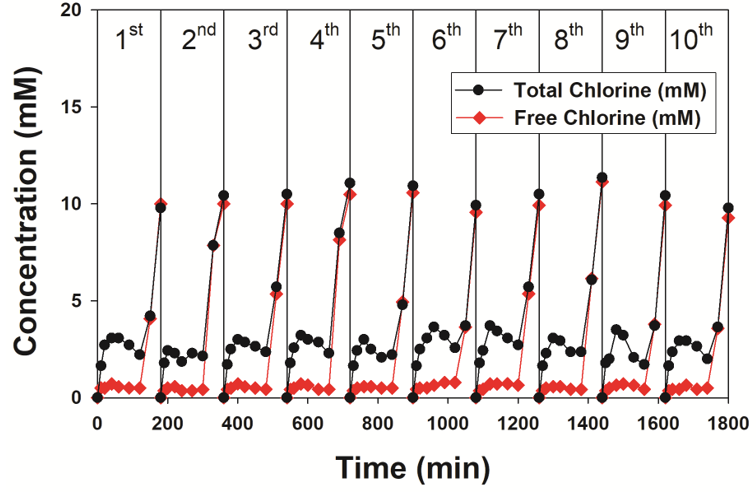


**Fig. S28** Degradation of ammonia on NFI/TiO_2_ for ten repeated runs. The concentration profile of total chlorine (black) and free chlorine (red) (Cathode: Pt, reference: Ag/AgCl, geometric surface area: 2 × 1 cm^2^, [Ammonia]_0_ = 25 mM, [Cl^-^]_0_ = 100 mM, and applied current density: 30 mA cm^-2^)


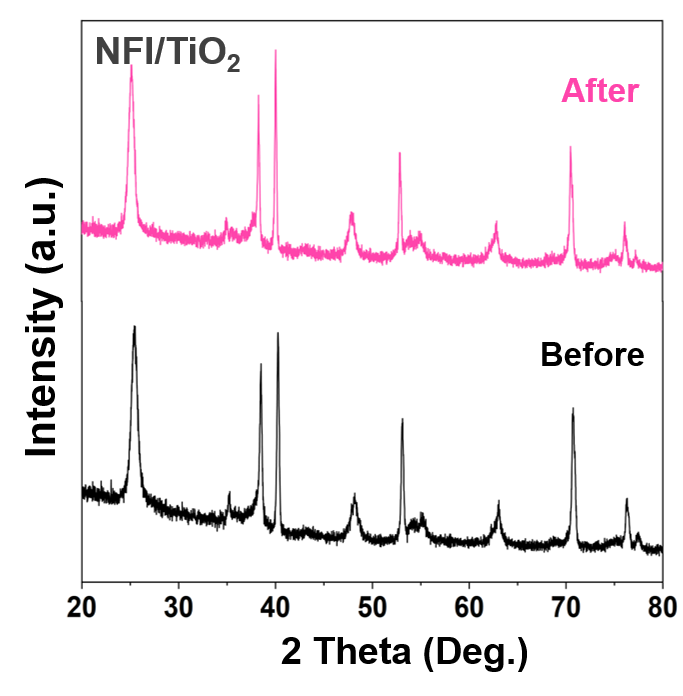


**Fig. S29** XRD pattern obtained from NFI/TiO_2_ before and after the batch electrolysis cycles

**
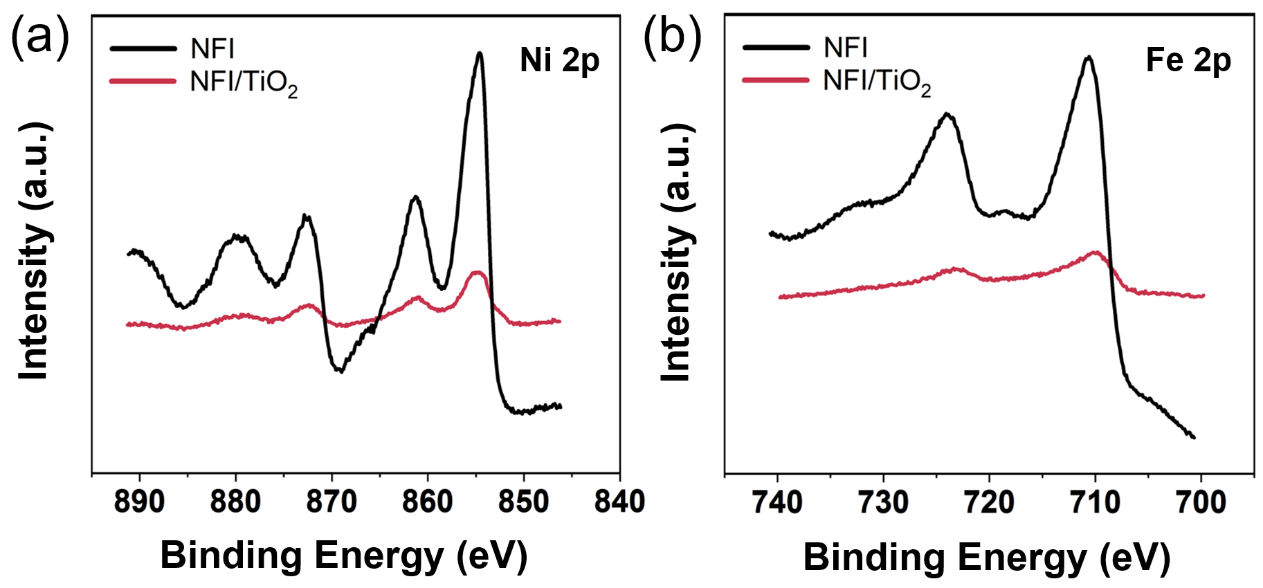
**

**Fig. S30** X-ray photoelectron spectra for the (**a**) Ni 2p and (**b**) Fe 2p of NFI and NFI/TiO_2_

**
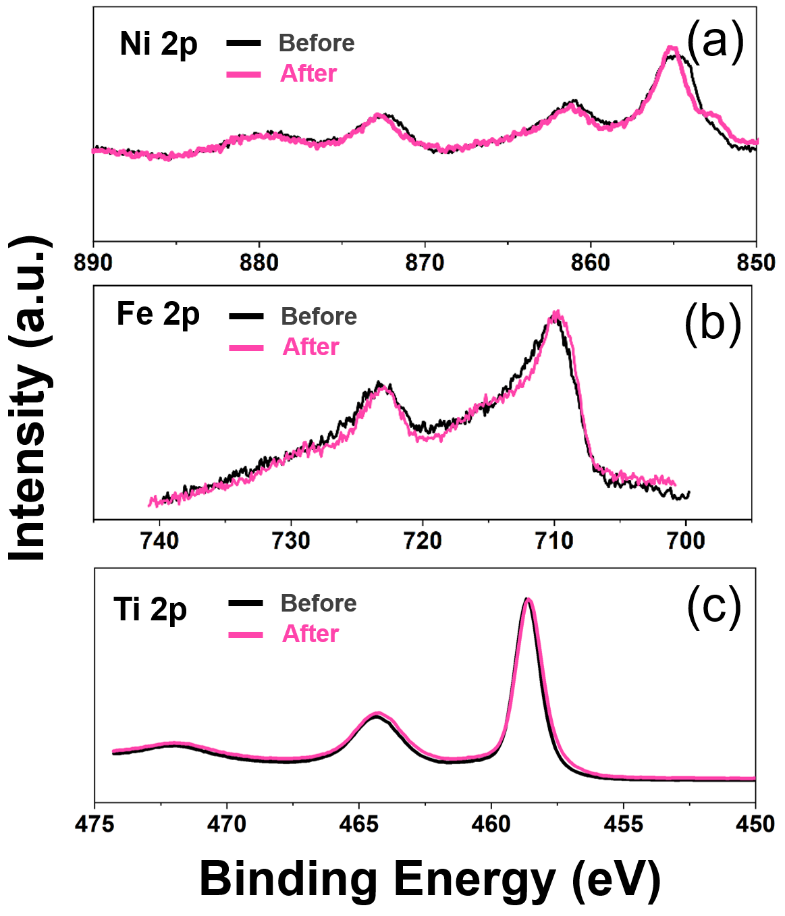
**

**Fig. S31** X-ray photoelectron spectra of (**a**) Ni 2p, (**b**) Fe 2p, and (**c**) Ti 2p of NFI/ TiO_2_ before and after the electrolysis

**
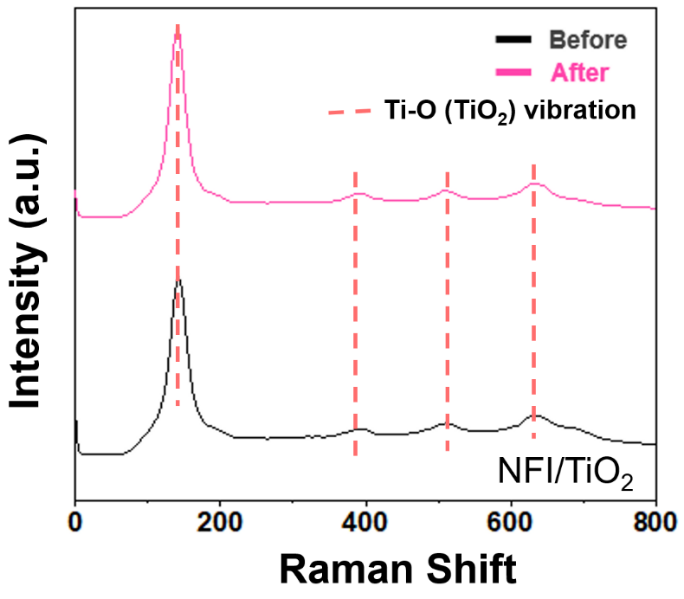
**

**Fig. S32** Raman spectra of NFI/TiO_2_ before and after electrolysis

*
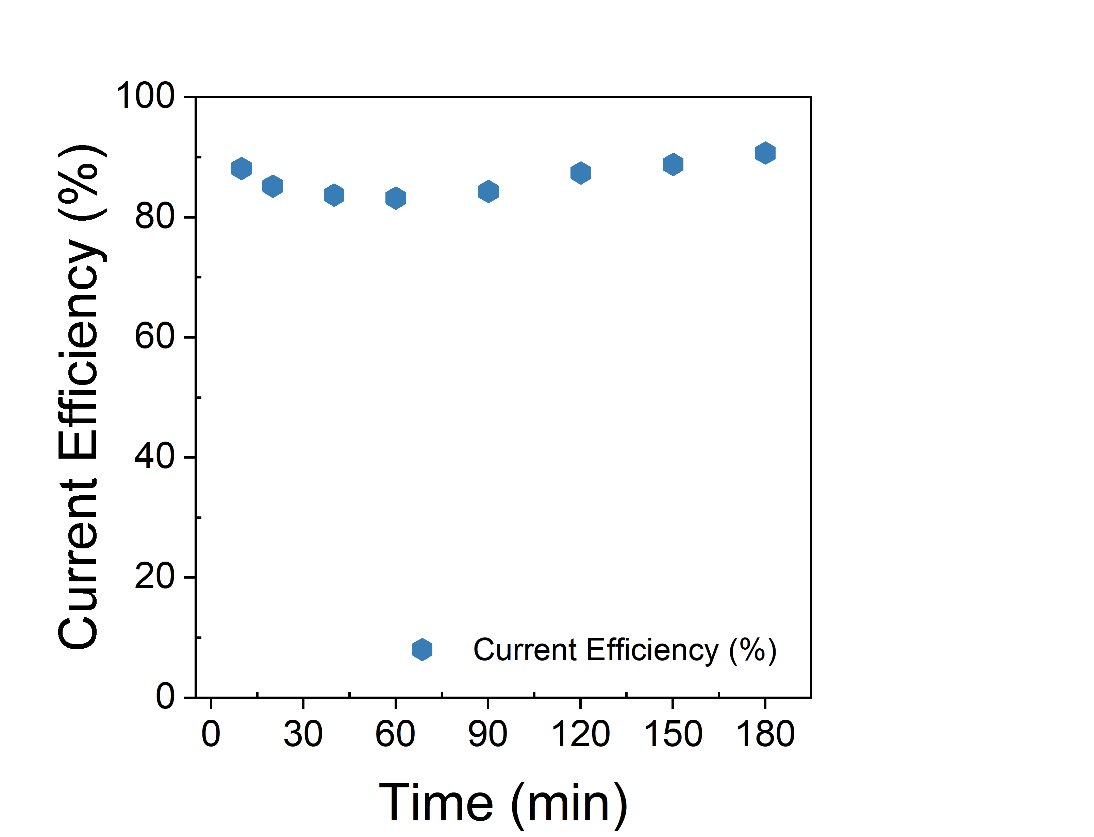
*

**Fig. S33** Current efficiency for HER as a function of time during the wastewater electrolysis

**Fig. S34** Fluorescence EEMs of the (a) influent and (b) effluent of the scaled up electrolysis cell for livestock wastewater treatment

**Supplementary References**

1. S. Mardani, M. Baghdadi, A. Torabian, B.A. Goharrizi, Electro-oxidation of ammonia using a continuous system equipped with RuO_2_@Ti mesh anode: Optimization of the design parameters with a focus on energy consumption and removal efficiency. Chem. Eng. Sci. **277** 118868 (2023) <https://doi.org/https://doi.org/10.1016/j.ces.2023.118868>
2. I. Salmerón, I. Oller, S. Malato, Electro-oxidation process assisted by solar energy for the treatment of wastewater with high salinity. Sci. Total Environ. **705** 135831 (2020) <https://doi.org/https://doi.org/10.1016/j.scitotenv.2019.135831>
3. O.T. Can, L. Gazigil, R. Keyikoglu, Treatment of intermediate landfill leachate using different anode materials in electrooxidation process. Environ. Prog. Sustainable Energy **41** e13722 (2022) <https://doi.org/https://doi.org/10.1002/ep.13722>
4. Q. Liang, Y. Gao, Z. Li, J. Cai, N. Chu et al., Electricity-driven ammonia oxidation and acetate production in microbial electrosynthesis systems. Front. Environ. Sci. Eng. **16** 1-10 (2022) <https://doi.org/https://doi.org/10.1007/s11783-021-1476-5>
5. S. Hong, G. Choi, N.T.Y. Phan, H. Shin, J. Lim, Efficient and durable iridium-doped SnO_2_ anode for reactive chlorine species-mediated urine wastewater treatment. Chem. Eng. J. 152698 (2024) <https://doi.org/https://doi.org/10.1016/j.cej.2024.152698>
6. G. Pérez, A. Fernández-Alba, A. Urtiaga, I. Ortiz, Electro-oxidation of reverse osmosis concentrates generated in tertiary water treatment. Water Res. **44** 2763-2772 (2010) <https://doi.org/https://doi.org/10.1016/j.watres.2010.02.017>
7. M. Zhou, L. Liu, Y. Jiao, Q. Wang, Q. Tan, Treatment of high-salinity reverse osmosis concentrate by electrochemical oxidation on BDD and DSA electrodes. Desalination **277** 201-206 (2011) <https://doi.org/https://doi.org/10.1016/j.desal.2011.04.030>
8. A.Y. Bagastyo, D.J. Batstone, I. Kristiana, B.I. Escher, C. Joll, J. Radjenovic, Electrochemical treatment of reverse osmosis concentrate on boron-doped electrodes in undivided and divided cell configurations. J. Hazard. Mater. **279** 111-116 (2014) <https://doi.org/https://doi.org/10.1016/j.jhazmat.2014.06.060>
9. J. Zou, X. Peng, M. Li, Y. Xiong, B. Wang, F. Dong, B. Wang, Electrochemical oxidation of COD from real textile wastewaters: kinetic study and energy consumption. Chemosphere **171** 332-338 (2017) <https://doi.org/https://doi.org/10.1016/j.chemosphere.2016.12.065>
10. N. Abdessamad, H. Akrout, L. Bousselmi, Anodic oxidation of textile wastewaters on boron-doped diamond electrodes. Environ. Technol. **36** 3201-3209 (2015) <https://doi.org/https://doi.org/10.1080/09593330.2015.1056235>
11. J. Yao, S. Lv, Z. Wang, L. Hu, J. Chen, Variation of current density with time as a novel method for efficient electrochemical treatment of real dyeing wastewater with energy savings. Environ. Sci. Pollut. Res. **29** 49976-49984 (2022) <https://doi.org/https://doi.org/10.1007/s11356-022-18927-3>
12. F. Medrano-Rodríguez, A. Picos-Benítez, E. Brillas, E.R. Bandala, T. Pérez, J.M. Peralta-Hernández, Electrochemical advanced oxidation discoloration and removal of three brown diazo dyes used in the tannery industry. J. Electroanal. Chem. **873** 114360 (2020) <https://doi.org/https://doi.org/10.1016/j.jelechem.2020.114360>
13. W. Can, H. Yao-Kun, Z. Qing, J. Min, Treatment of secondary effluent using a three-dimensional electrode system: COD removal, biotoxicity assessment, and disinfection effects. Chem. Eng. J. **243** 1-6 (2014) <https://doi.org/https://doi.org/10.1016/j.cej.2013.12.044>
14. K. Chandrasekaran, H. Selvaraj, M. Sundaram, Electrochemical oxidation with the aerobic pretreatment process for sulfate-rich tannery effluent. Environ. Sci. Pollut. Res. **26** 12194-12204 (2019) <https://doi.org/https://doi.org/10.1007/s11356-019-04614-3>
15. D. Ozturk, A.E. Yilmaz, Treatment of slaughterhouse wastewater with the electrochemical oxidation process: Role of operating parameters on treatment efficiency and energy consumption. J. Water Process Eng. **31** 100834 (2019) <https://doi.org/https://doi.org/10.1016/j.jwpe.2019.100834>
16. A.J. dos Santos, H. Shen, M.R. Lanza, Q. Li, S. Garcia-Segura, Electrochemical oxidation of surfactants as an essential step to enable greywater reuse. Environ. Technol. Innovation 103563 (2024) <https://doi.org/https://doi.org/10.1016/j.eti.2024.103563>
17. Z. Lang, M. Zhou, Q. Zhang, X. Yin, Y. Li, Comprehensive treatment of marine aquaculture wastewater by a cost-effective flow-through electro-oxidation process. Sci. Total Environ. **722** 137812 (2020) <https://doi.org/https://doi.org/10.1016/j.scitotenv.2020.137812>
18. X. Yunqing, L. Jianwei, Application of electrochemical treatment for the effluent from marine recirculating aquaculture systems. Procedia Environ. Sci. **10** 2329-2335 (2011) <https://doi.org/https://doi.org/10.1016/j.proenv.2011.09.363>
19. V. Díaz, R. Ibáñez, P. Gómez, A. Urtiaga, I. Ortiz, Kinetics of electro-oxidation of ammonia-N, nitrites and COD from a recirculating aquaculture saline water system using BDD anodes. Water Res. **45** 125-134 (2011) <https://doi.org/https://doi.org/10.1016/j.watres.2010.08.020>
20. C. Zhang, D. He, J. Ma, T.D. Waite, Active chlorine mediated ammonia oxidation revisited: Reaction mechanism, kinetic modelling and implications. Water Res. **145** 220-230 (2018) <https://doi.org/https://doi.org/10.1016/j.watres.2018.08.025>
21. Y. Ruan, C. Lu, X. Guo, Y. Deng, S. Zhu, Electrochemical treatment of recirculating aquaculture wastewater using a Ti/RuO_2_-IrO_2_ anode for synergetic total ammonia nitrogen and nitrite removal and disinfection. Trans. ASABE **59** 1831-1840 (2016) <https://doi.org/https://doi.org/10.13031/trans.59.11630>
22. R. Ben-Asher, O. Lahav, Minimization of THM formation in seawater-fed recirculating aquaculture systems operated with electrochemical NH_4_^+^ removal. Aquaculture **502** 162-175 (2019) <https://doi.org/https://doi.org/10.1016/j.aquaculture.2018.12.025>
23. X. He, Z. Chai, F. Li, C. Zhang, D. Li et al., Advanced treatment of biologically pretreated coking wastewater by electrochemical oxidation using Ti/RuO_2_–IrO_2_ electrodes. J. Chem. Technol. Biotechnol. **88** 1568-1575 (2013) <https://doi.org/https://doi.org/10.1002/jctb.4006>
24. A. Anglada, R. Ibanez, A. Urtiaga, I. Ortiz, Electrochemical oxidation of saline industrial wastewaters using boron-doped diamond anodes. Catal. Today **151** 178-184 (2010) <https://doi.org/https://doi.org/10.1016/j.cattod.2010.01.033>
